# Sec13 promotes oligodendrocyte differentiation and myelin repair through autocrine pleiotrophin signaling

**DOI:** 10.1172/JCI155096

**Published:** 2022-04-01

**Authors:** Zhixiong Liu, Minbiao Yan, Wanying Lei, Rencai Jiang, Wenxiu Dai, Jialin Chen, Chaomeng Wang, Li Li, Mei Wu, Ximing Nian, Daopeng Li, Di Sun, Xiaoqi Lv, Chaoying Wang, Changchuan Xie, Luming Yao, Caiming Wu, Jin Hu, Naian Xiao, Wei Mo, Zhanxiang Wang, Liang Zhang

**Affiliations:** 1 Department of Neuroscience, Institute of Neurosurgery, and Department of Neurosurgery, The First Affiliated Hospital, State Key Laboratory of Cellular Stress Biology, School of Medicine,; 2 Xiamen Key Laboratory of Brain Center, The First Affiliated Hospital,; 3 School of Life Sciences, Innovation Center for Cell Signaling Network, and; 4 Department of Neurology, The First Affiliated Hospital, Xiamen University, Fujian, China.

**Keywords:** Cell Biology, Neuroscience, Demyelinating disorders, Multiple sclerosis, Protein traffic

## Abstract

Dysfunction of protein trafficking has been intensively associated with neurological diseases, including neurodegeneration, but whether and how protein transport contributes to oligodendrocyte (OL) maturation and myelin repair in white matter injury remains unclear. ER-to-Golgi trafficking of newly synthesized proteins is mediated by coat protein complex II (COPII). Here, we demonstrate that the COPII component Sec13 was essential for OL differentiation and postnatal myelination. Ablation of Sec13 in the OL lineage prevented OPC differentiation and inhibited myelination and remyelination after demyelinating injury in the central nervous system (CNS), while improving protein trafficking by tauroursodeoxycholic acid (TUDCA) or ectopic expression of COPII components accelerated myelination. COPII components were upregulated in OL lineage cells after demyelinating injury. Loss of Sec13 altered the secretome of OLs and inhibited the secretion of pleiotrophin (PTN), which was found to function as an autocrine factor to promote OL differentiation and myelin repair. These data suggest that Sec13-dependent protein transport is essential for OL differentiation and that Sec13-mediated PTN autocrine signaling is required for proper myelination and remyelination.

## Graphical abstract

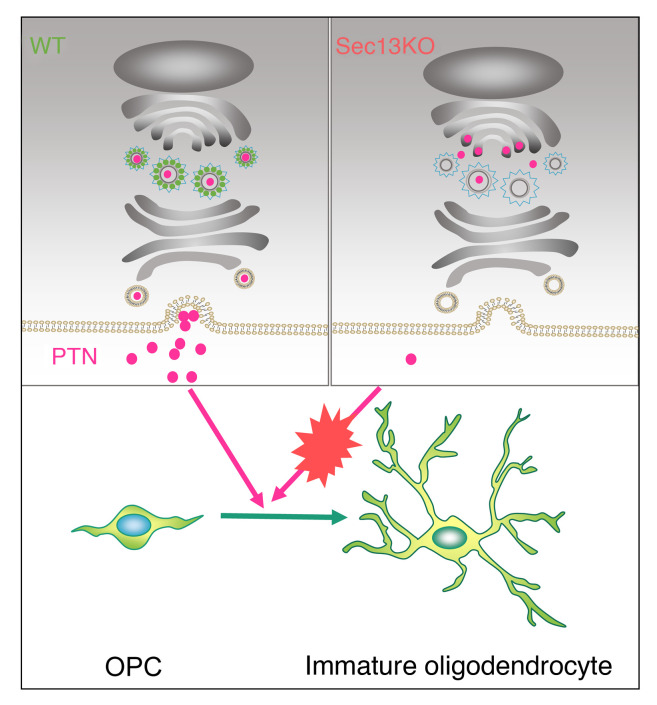

## Introduction

Myelination in the central nervous system (CNS) by oligodendrocytes (OLs) is essential for rapid impulse conduction and normal brain function (1, 2). Disrupted myelin repair impairs nerve conduction and contributes to neurological dysfunction, axon degeneration, and progression of diseases such as multiple sclerosis (MS) and leukodystrophies (3). Oligodendrocyte progenitor cells (OPCs) are present in demyelinated regions of patients with MS, and there is evidence of impaired OL differentiation (4). Therefore, understanding how OL differentiation and remyelination are regulated has implications for proper brain functions and therapy in demyelinating diseases.

Disrupted protein transport and abnormal protein aggregation have been frequently observed and studied in neurological diseases (5). Trafficking of transmembrane and soluble proteins from the endoplasmic reticulum (ER) to the Golgi organelle is mediated by coat protein complex II (COPII) (6). COPII is composed of 5 core components, including a small GTPase, SAR1, and 2 cytosolic protein complexes, Sec23-Sec24 and Sec13-Sec31 (6). Dysregulation of COPII components has been reported to inhibit protein secretion and affect cell differentiation, function, and homeostasis (7–12). Increasing evidence has indicated the connections between COPII components and human neurological disorders. Sec24B variants induce neural tube defects (13). Mutant Sec31A causes a severe neurological syndrome (14). During OL differentiation, OLs undergo remarkable process extension and membrane expansion, which is accompanied by a large amount of protein trafficking (15). However, a comprehensive understanding of the physiological function and the underlying mechanism of COPII components in OL differentiation and myelination is missing.

Both extrinsic environmental signals and intrinsic signaling pathways play important roles in regulating OL differentiation (16). Among extracellular cues, the majority of them are paracrine factors, released from other cells, such as astrocytes (17, 18). Much less is known about whether OL differentiation is also regulated by autocrine signaling. Here, we found that COPII components were upregulated after demyelinating injury. Ectopic expression of these components promoted OL differentiation, whereas knockdown of these components impaired myelin gene transcription. Ablation of the COPII component Sec13 prevented OPC differentiation and myelination by inhibiting pleiotrophin (PTN) secretion. Mechanistically, autocrine PTN signaling promoted OL maturation by binding to the PTPRZ receptor and activating p190RhoGAP signaling. Moreover, exogenous expression of PTN accelerated remyelination after demyelination injury. These findings suggest that the COPII component Sec13 is required for OL differentiation and that Sec13-mediated autocrine PTN signaling plays an important role in CNS myelination.

## Results

### The COPII complex is implicated in remyelination after demyelination.

**Figure 1:**
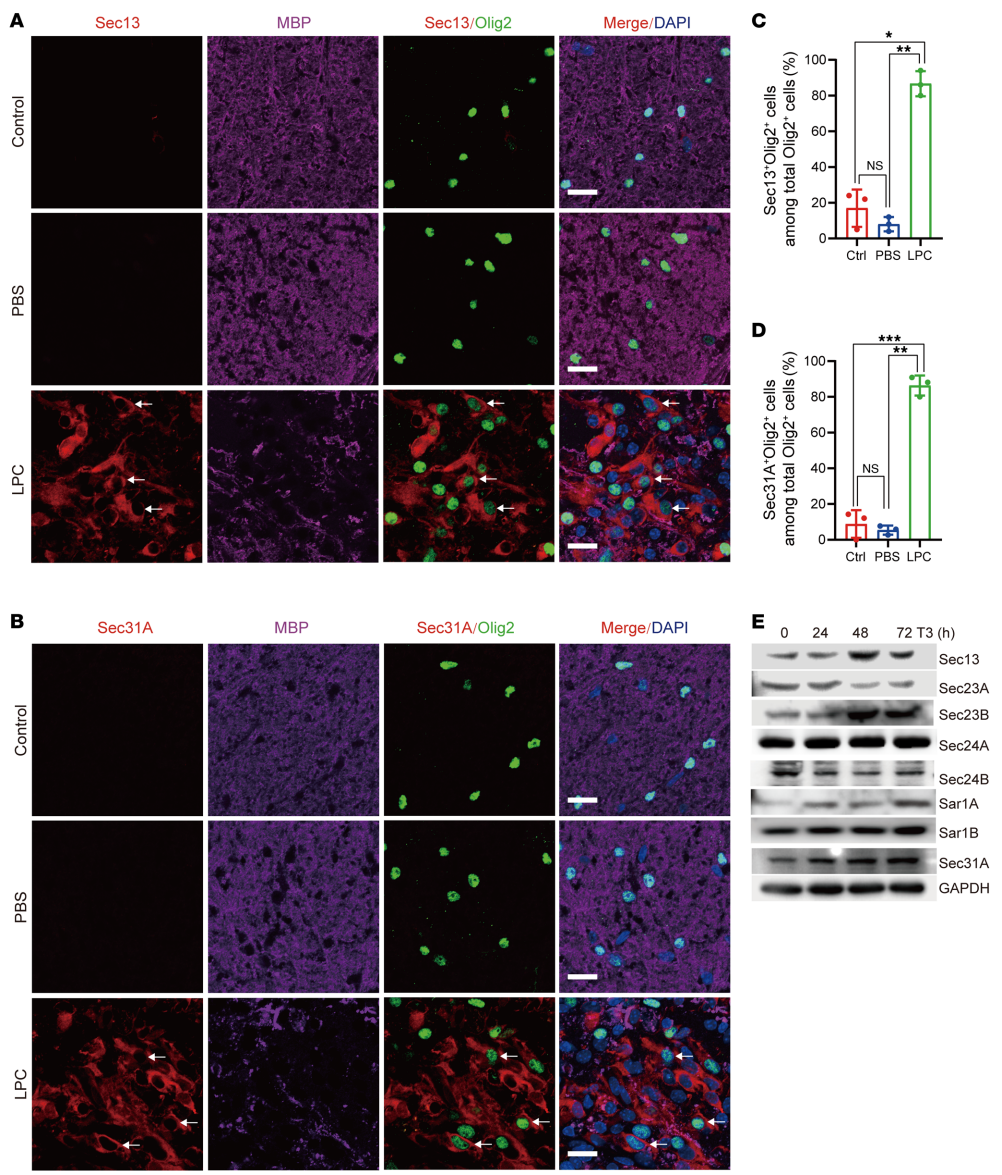
The COPII complex is implicated in remyelination after demyelination. (**A**) Immunostaining for Sec13, Olig2, and MBP at 7 dpl in nonlesion white matter control, PBS-injected, and LPC-injured spinal cords from 8-week-old mice. Arrows indicate Sec13^+^ and Olig2^+^ cells. Scale bars: 20 μm. (**B**) Immunostaining for Sec31A, Olig2, and MBP at 7 dpl in nonlesion white matter control, PBS-injected, and LPC-injured spinal cords from 8-week-old mice. Arrows indicate Sec31A^+^ and Olig2^+^ cells. Scale bars: 20 μm. (**C** and **D**) Quantification of Sec13A^+^Olig2^+^ cells among Olig2^+^ cells (**C**) and Sec31A^+^Olig2^+^ cells among Olig2^+^ cells (**D**) in LPC lesion sites (*n =* 3 animals/treatment). Data represent the mean ± SD. *P < 0.05, **P < 0.01, and ***P < 0.001, by 1-way ANOVA with Tukey’s correction for multiple comparisons. (**E**) Immunoblotting of the indicated proteins in cultured rat OPCs and differentiating OLs after T3 treatment. Ctrl, control.

**Figure 2:**
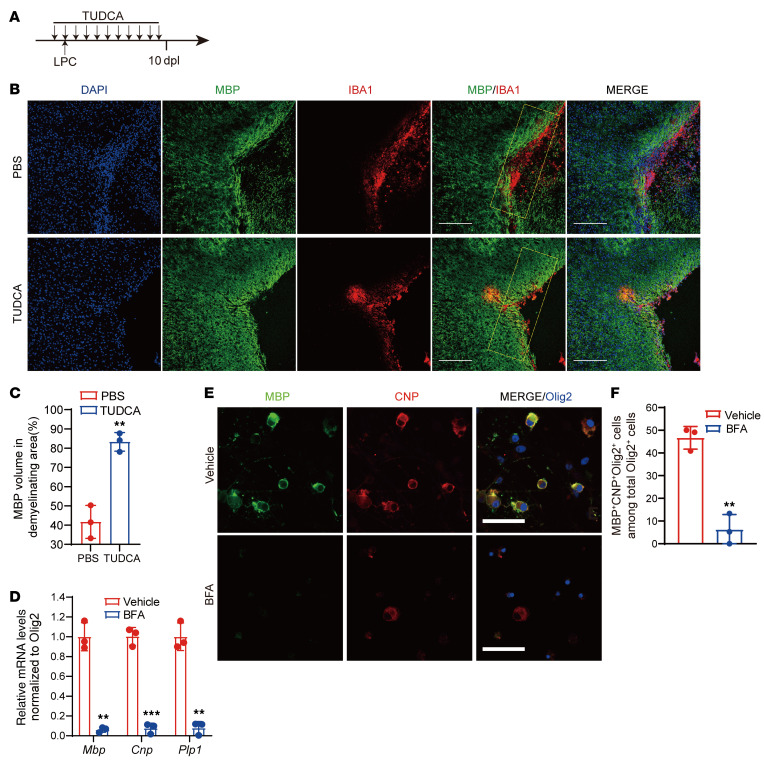
Protein transport is necessary for myelination. (**A**) Diagram showing TUDCA administration and the LPC injection schedule. (**B**) Immunostaining for MBP and IBA1 in corpus callosum lesions of control and TUDCA-treated mice at 10 dpl. Scale bars: 200 μm. (**C**) Quantification of MBP^+^ areas of demyelinating regions in corpus callosum from control and TUDCA-treated mice at 10 dpl (*n =* 3 animals/treatment). (**D**) Real-time PCR analysis of myelination-associated genes in primary rat OPCs under differentiation conditions in the presence or absence of BFA (0.5 g/μL). BFA was applied to cells when switching to differentiation media and incubation for 48 hours (*n =* 3 independent experiments). (**E**) Coimmunostaining for MBP, CNP, and Olig2 in primary rat OPCs under differentiation conditions for 72 hours in the presence or absence of BFA (0.5 μg/μL). Scale bars: 50 μm. (**F**) Quantification of MBP^+^CNP^+^Olig2^+^ cells as a percentage of total Olig2^+^ cells after 3 days of differentiation (*n =* 3 independent experiments). Data represent the mean ± SD. **P < 0.01 and ***P < 0.001, by 2-tailed, unpaired Student’s *t* test.

OPCs exist in the adult mouse CNS and can differentiate into mature OLs to mediate adult myelinogenesis, which is important for remyelination following demyelinating injury (19, 20). Focal injection of lysolecithin (LPC) in the white matter causes acute demyelinating injury followed by myelin regeneration (21). We found that expression of the COPII components Sec13 and Sec31A was hardly detectable in non-lesion regions of adult spinal white matter and that focal injection of PBS did not affect their expression. However, expression of Sec13 and Sec31A was significantly upregulated following demyelination, and it was substantially expressed in the OL lineage as indicated by Olig2-expressing cells (Figure 1, A–D), suggesting that protein trafficking mediated by COPII may play important roles during the remyelination process. Similar findings were also observed in an ethidium bromide–induced (EB-induced) demyelination assay (Supplemental Figure 1, A and B; supplemental material available online with this article; https://doi.org/10.1172/JCI155096DS1). We further analyzed the expression patterns of COPII components during OL differentiation. Sec13, Sec23B, Sar1A, Sar1B, and Sec31A were upregulated in response to differentiation cues in vitro (Figure 1E and Supplemental Figure 1C). These data indicate that COPII-mediated protein transport might be important for remyelination in the CNS. Therefore, we sought to determine whether tauroursodeoxycholic acid (TUDCA), which is a chemical chaperone and was shown to ameliorate protein transport (22, 23), could accelerate remyelination after LPC-induced demyelinating injury. Surprisingly, 10 days post lesion (dpl), we found that myelin repair was significantly accelerated in the corpus callosum region of mice treated with 500 mg/kg TUDCA once a day for 11 days as indicated with myelin basic protein (MBP) staining (Figure 2, A–C). Although the number of PDGFRα^+^ OPCs at the lesion site was slightly decreased after TUDCA treatment (Supplemental Figure 2, A and B), lesion size or the number of Olig2^+^ OLs at the lesion site was not affected (Supplemental Figure 2, C–E). In addition, treatment with the ER-Golgi trafficking inhibitor brefeldin A (BFA) strongly inhibited the expression of myelin genes (Figure 2, D–F). Collectively, these findings indicate that COPII-mediated protein transport is implicated in OL differentiation and remyelination.

### The COPII component Sec13 is required for myelination in the CNS.

**Figure 3:**
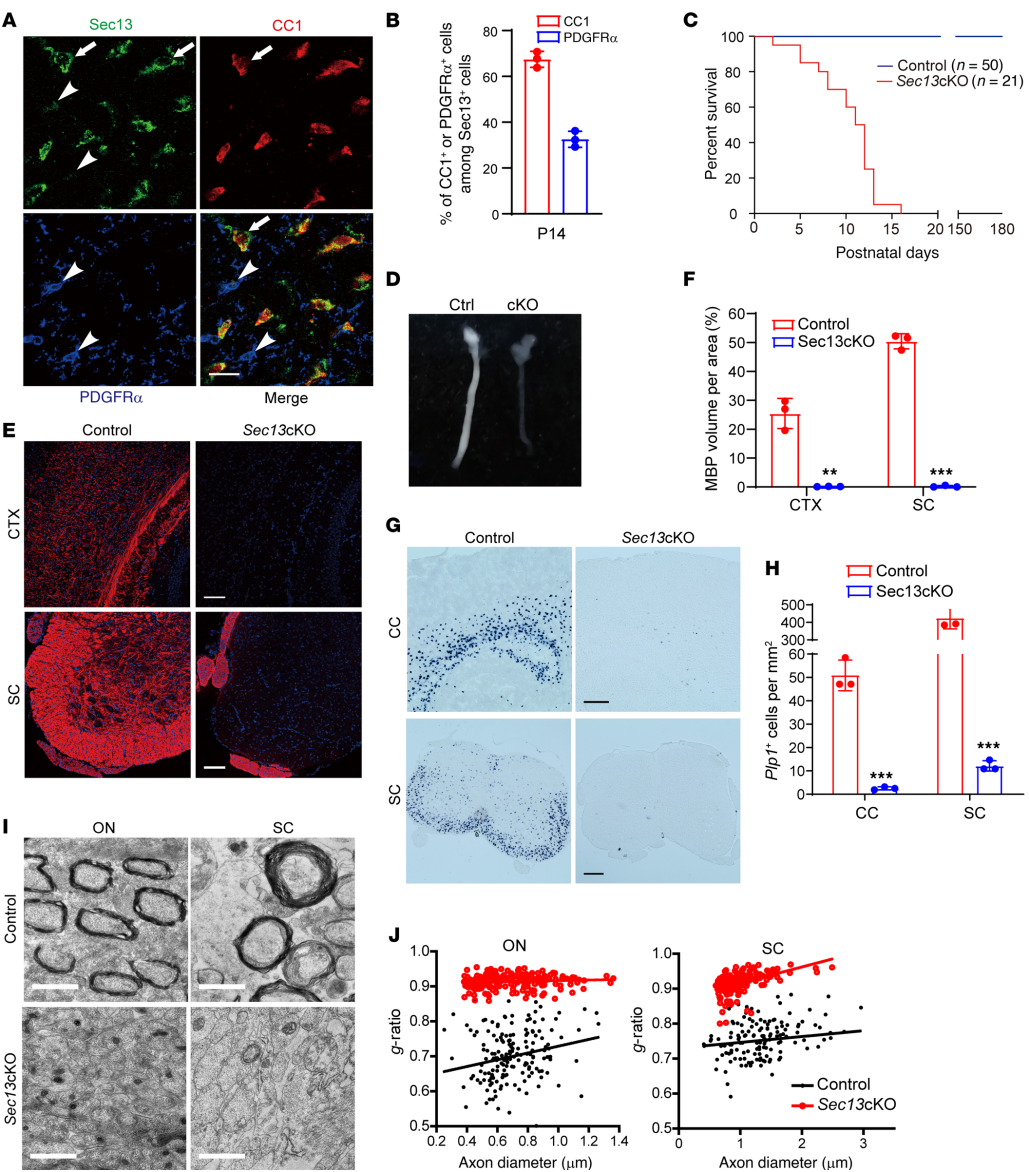
The COPII component Sec13 is required for myelination in the CNS. (**A**) Coimmunostaining for Sec13, PDGFRα, and CC1 in the corpus callosum of WT mice at P14. Arrows indicate Sec13^+^and CC1^+^ cells; arrowheads indicate Sec13^+^ and PDGFRα^+^ cells. Scale bars: 20 μm. (**B**) Quantification of CC1^+^ or PDGFRα^+^ cells among Sec13^+^ cells in the corpus callosum at P14 (*n =* 3 WT mice). (**C**) Survival curves for control and *Sec13*-cKO mice (*n =* 50 control and *n* = 21 mutant mice). (**D**) Representative images of optic nerves from control and *Sec13*-cKO mice at P12. (**E**) Immunostaining for MBP in the cortex (CTX) and spinal cord (SC) of control and *Sec13*-cKO mice at P14. Nuclei were stained with DAPI. Scale bars: 100 μm. (**F**) Quantification of MBP^+^ volume in the cortex (CTX) and spinal cord (SC) of control and *Sec13*-cKO mice at P14 (*n =* 3 control and *n* = 3 mutant animals). (**G** and **H**) ISH (**K**) and quantification (**L**) of *Plp1* in the corpus callosum (CC) and spinal cord of control and *Sec13*-cKO mice at P14 (*n =* 3 control and *n* = 3 mutant animals). Scale bars: 250 μm. (**I**) Electron micrographic analysis of optic nerves (ON) and spinal cord (SC) in control and *Sec13*-cKO mice at P14. Scale bars: 1 μm. (**J**) Myelin *g*-ratio in optic nerves and spinal cords of control and *Sec13*-cKO mice at P14. Data represent the mean ± SD. **P < 0.01 and ***P < 0.001, by 2-tailed, unpaired Student’s *t* test.

To further gain insights into the functions of COPII during OL development, we respectively attenuated their expression in primary rat OPCs using siRNAs (Supplemental Figure 3A). Knockdown of COPII components individually inhibited the expression of myelin-associated genes, including 2′,3′-cyclic nucleotide 3′-phosphodiesterase (*Cnp*) and proteolipid protein (*Plp1*) (Supplemental Figure 3B), suggesting that COPII was critical for proper OL differentiation in vitro. Sec13 needs to cooperate with Sec31 to promote COPII vesicle budding and protein secretion (24, 25). In agreement with this, cotransfection of Sec13 and Sec31A, but not Sec13 or Sec31A alone, promoted myelin gene transcription and CNP protein expression (Supplemental Figure 3, C and D). Unlike other COPII components, there is only one Sec13 isoform, and knockdown of Sec13 impairs cell differentiation, thus we focused on Sec13 function in OL development. Sec13 was strongly expressed in CC1^+^ mature OLs, but weakly in PDGFRα^+^ OPCs in the P14 corpus callosum (Figure 3A). The proportions of CC1^+^ cells among Sec13^+^ cells in the corpus callosum at P14 were 66%, suggesting that Sec13 is largely restricted to differentiating OLs in the OL lineage (Figure 3B). Sec13 expression was also observed in NeuN^+^ neurons in the cortex adjacent to the corpus callosum, but was hardly detectable in glial fibrillary acidic protein–positive (GFAP^+^) astrocytes or IBA1^+^ microglia in the corpus callosum at P14 (Supplemental Figure 3E). Similarly, we detected other COPII components, Sar1B and Sec23A, in Olig2^+^ OLs and NeuN^+^ neurons, but not in GFAP^+^ astrocytes, or IBA1^+^ microglia (Supplemental Figure 4, A and B). Consistent with previous observations (26), Sec13 was primarily located at the ER exit site (ERES), where it colocalized with Sec24A in early differentiating immature OLs (Supplemental Figure 4C). To assess the role of Sec13 in OL development in vivo, we crossed Sec13-hypomorphic mice with FLPe-expressing transgenic mice to remove the NEO cassette (Supplemental Figure 4, D and E, and ref. 27). The resulting *Sec13^fl/fl^* mice had expression levels comparable to those of WT mice (Supplemental Figure 4F). We then selectively deleted Sec13 in the OL lineage by crossing *Sec13^fl/fl^* mice with an Olig1-Cre mouse line with directed Cre expression in the OL lineage cells (28), including OPCs and mature myelinating OLs in the CNS. Sec13 protein expression in Olig2^+^ OLs was substantially reduced in the spinal cord and corpus callosum of *Sec13*–conditional knockout mice (*Sec13* *^fl/fl^* *Olig1-Cre*^+/–^, referred to herein as *Sec13*-cKO mice) at P14 (Supplemental Figure 5, A–D). In contrast, Sec13 expression was not affected in NeuN^+^ neurons (Supplemental Figure 5, E and F). The resulting *Sec13*-cKO mutant mice were born at Mendelian ratios and appeared to be normal at birth, but developed severe tremor and seizures, reminiscent of myelin-deficient mice, and died at postnatal week 2, in contrast to the normal lifespan of WT and heterozygotic mice (Figure 3C). The myelinating optic nerve from *Sec13*-cKO mice at P12 was translucent compared with that of the control, indicating severe hypomyelination (Figure 3D). Indeed, the expression of myelin markers such as MBP and *Plp* was significantly diminished in the spinal cord and corpus callosum of mutant mice at P14 (Figure 3, E–H, and Supplemental Figure 6A). We also assessed the extent of myelination by staining with FluoroMyelin and observed a decrease of myelination in the corpus callosum of mutant mice at P14 (Supplemental Figure 6B). Myelination deficits were further confirmed by electron microscopy. Myelinated axons were hardly detectable in the optic nerve or spinal cord of mutant mice at P14, in contrast to the large number of myelinated axons that were observed in control mice (Figure 3I). The thickness of myelin sheaths around axons assessed by g-ratios was substantially reduced in mutant mice (Figure 3J). Together, these data suggest that the COPII component Sec13 is required for CNS myelination.

Since Sec31A cooperated with Sec13 to promote myelin gene transcription and was upregulated upon LPC injury, we therefore tested the function of Sec31A in developmental myelination. Mice were injected with modified antisense oligonucleotides (ASOs) against Sec31A mRNA on P3 and harvested on P14. The MBP signal intensity was remarkably decreased in the brains of ASO-Sec31A–injected mice compared with signal intensity in control mice (Supplemental Figure 6, C and D).

### Sec13 is required for OPC differentiation.

**Figure 4:**
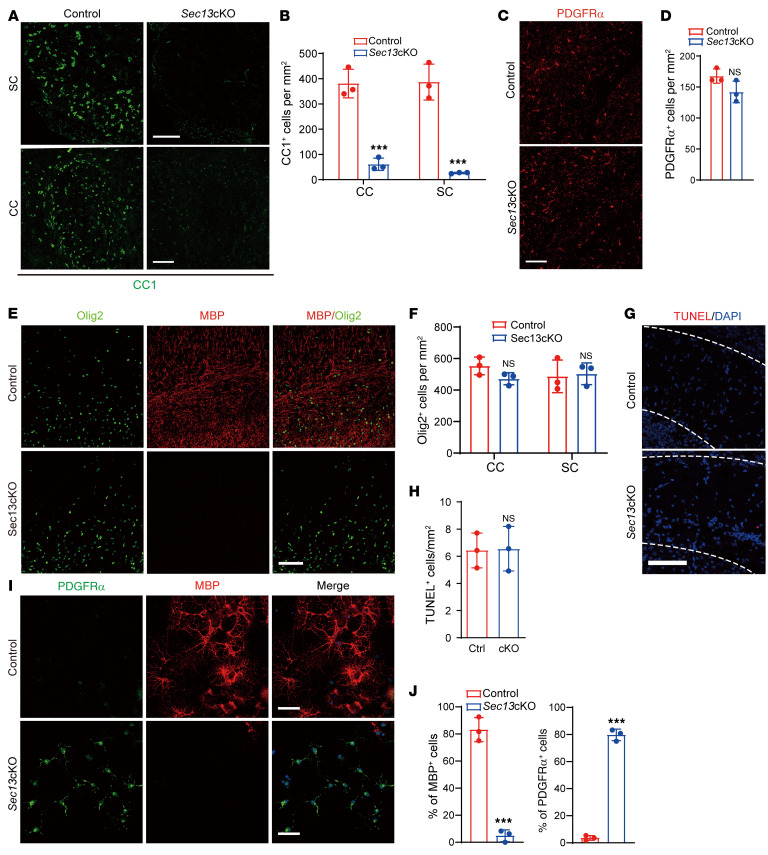
Sec13 is required for OPC differentiation. (**A** and **B**) Immunostaining (**A**) and quantification (**B**) of CC1 expression in the spinal cord and corpus callosum of control and *Sec13*-cKO mice at P14 (*n =* 3 control and *n* = mutant animals. Scale bars: 100 μm. (**C** and **D**) Immunostaining (**C**) and quantification (**D**) of PDGFRα in the corpus callosum of control and *Sec13*-cKO mice at P14 (*n =* 3 control and *n* = 3 mutant animals). Scale bars: 100 μm. (**E** and **F**) Immunostaining (**E**) and quantification (**F**) of Olig2^+^ cells in the corpus callosum and spinal cord of control and *Sec13*-cKO mice at P14 (*n =* 3 control and *n* = 3 mutant animals). Scale bars: 100 μm. (**G** and **H**) Representative images (**G**) and quantification (**H**) of TUNEL signals per field (1 mm^2^) in the corpus callosum of control and *Sec13*-cKO mice at P7 (*n =* 3 control and *n* = 3 mutant animals). Scale bar: 100 μm. (**I**) Immunolabeling for PDGFRα and MBP in control and *Sec13*-cKO primary OPCs under differentiation conditions for 96 hours. Scale bars: 50 μm. (**J**) Quantification of MBP^+^ and PDGFRα^+^ cells from control and *Sec13*-cKO mice under differentiation conditions for 96 hours (*n =* 3 control and *n* = 3 mutant animals). Data represent the mean ± SD. ***P < 0.001, by 2-tailed, unpaired Student’s *t* test.

The hypomyelination phenotype detected in Sec13-mutant mice could be due to arrested OPC maturation. To test this hypothesis, we analyzed OPC and OL numbers. In contrast with the marked reduction of CC1^+^ OLs, the total numbers of PDGFRα^+^ OPCs were comparable between control and *Sec13*-cKO mutants mice (Figure 4, A–D). Loss of Sec13 did not appear to change the number of Olig2^+^ OLs (Figure 4, E and F). We did not detect any significant increase in apoptotic cells by TUNEL assay (Figure 4, G and H). Meanwhile, the Sec13 mutants did not exhibit significant alteration of astrocytes, neurons, or axons identified by GFAP, NeuN, and NF200 staining (Supplemental Figure 7, A and B). These data suggest that ablation of Sec13 prevents OPCs from differentiation. To further determine whether defects in OL maturation in *Sec13*-cKO animals are cell autonomous because of *Sec13* deletion, we purified OPCs by immunopanning from the neonatal brain for in vitro studies. Loss of Sec13 resulted in a significant decrease in the number of MBP^+^ or CNP^+^ OLs after triiodothyronine (T3) treatment, whereas the cells remained in a PDGFR-expressing state (Figure 4, I and J, and Supplemental Figure 7, C and D). Consistently, we found that expression of myelin-associated gene transcripts was also significantly inhibited (Supplemental Figure 7E). In addition, a reduction of Sec13 with siRNA-mediated knockdown in rat OPCs resulted in a similar decrease in the number of MBP^+^ OLs. In contrast, control OPCs readily differentiated into MBP^+^ OLs (Supplemental Figure 7, F–H). Furthermore, reintroduction of siRNA-resistant Sec13 could rescue the defects in myelin gene expression (Supplemental Figure 7I). To further examine the effects of Sec13 inactivation on OPC differentiation during early postnatal development in a time-controlled manner, we generated OPC-inducible *Sec13* mutants by crossing *Sec13 ^fl/fl^* mice with *PDGFRα-CreERT* mice and inducing recombination of Sec13 in OPCs at P3 by tamoxifen administration (Supplemental Figure 8A). We observed effective depletion of Sec13 in Olig2^+^ OLs (Supplemental Figure 8, B and C), and expression of MBP in *PDGFRα-CreERT* *Sec13^fl/fl^* mutant (*Sec13*-iKO) mice was reduced compared with that in control littermates at P12 (Supplemental Figure 8, D and E). Together, these findings suggest that Sec13 is required for OPC differentiation.

### Sec13 is critical for adult remyelination after demyelination.

**Figure 5:**
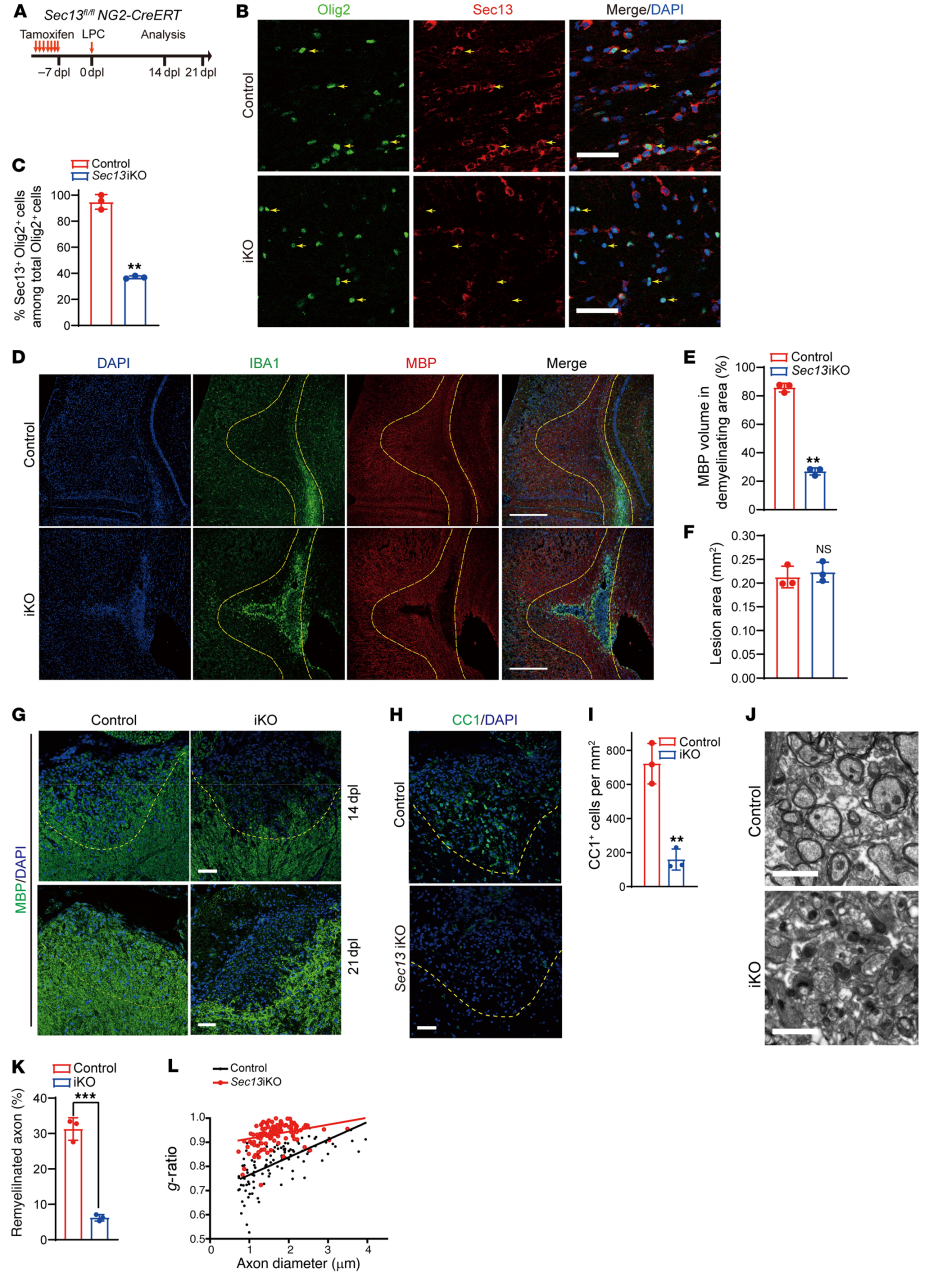
Sec13 is critical for adult remyelination after demyelination. (**A**) Diagram showing tamoxifen administration to 8-week-old control (*Sec13 ^fl/fl^*) and *Sec13*-iKO (*Sec13 ^fl/fl^* *NG2-CreERT*) mice followed by LPC injection 7 days later. (**B**) Immunostaining for Olig2 and Sec13 in the corpus callosum of control and *Sec13*-iKO mice at 14 dpl. Arrowheads indicate Olig2^+^ cells. Scale bars: 50 μm. (**C**) Quantification of Sec13^+^Olig2^+^ cells as a percentage of total Olig2^+^ cells in the corpus callosum at 14 dpl (*n =* 3 control and *n* = 3 mutant animals). (**D**) Immunostaining for IBA1 and MBP in corpus callosum lesions of control and *Sec13*-iKO mice at 14 dpl. Scale bars: 200 μm. (**E** and **F**) Quantification of MBP^+^ volume (**E**) and lesion area (**F**) in corpus callosum lesions of control and *Sec13*-iKO mice at 14 dpl (*n =* 3 control and *n* = 3 mutant animals). (**G**) Immunostaining for MBP in spinal cord lesions of control and *Sec13*-iKO mice at 14 dpl and 21 dpl. Scale bars: 50 μm. (**H** and **I**) Immunostaining (**H**) and quantification (**I**) of CC1^+^ cells in spinal cord lesions of control and *Sec13*-iKO mice at 14 dpl (*n =* 3 control and 3 mutant animals). Scale bar: 50 μm. (**J**) Electron microscopic images of LPC-induced lesions in spinal cords from control and *Sec13*-iKO mice at 14 dpl. Scale bars: 2 μm. (**K**) Quantification of remyelinated axons in LPC-induced lesions in spinal cords from control and *Sec13*-iKO mice at 14 dpl (*n =* 3 control and *n* = 3 mutant animals). (**L**) Myelin *g*-ratios in LPC-induced lesions of spinal cord from control and *Sec13*-iKO mice at 14 dpl. Data represent the mean ± SD. **P < 0.01 and ***P < 0.001, by 2-tailed, unpaired Student’s *t* test.

Given the essential role of Sec13 in developmental myelination, we hypothesized that Sec13 was required for remyelination after injury. We administered tamoxifen by oral gavage prior to LPC injection into 8-week-old control (*Sec13^fl/fl^*) and *Sec13*-iKO (*Sec13^fl/fl^ NG2-CreERT*) mice (Figure 5A), which induced effective depletion of Sec13 in Olig2^+^ OLs (Figure 5, B and C). At 14 dpl and 21 dpl, MBP expression was substantially reduced in the corpus callosum and spinal cord of *Sec13*-iKO mice compared with expression in control mice (Figure 5, D–G), whereas the lesion area was similar between control and iKO mice (Figure 5F and Supplemental Figure 9A). Consistent with these findings, fewer CC1^+^ differentiating OLs were detected in the lesions of *Sec13*-iKO corpus callosum and spinal cord than in controls (Figure 5, H and I, and Supplemental Figure 9, B and C). In contrast, depletion of Sec13 did not appear to impair the recruitment of PDGFRα^+^ OPCs or their proliferation (Supplemental Figure 9, D and E, and ref. 29). Quantification of Olig2^+^ or PDGFRα^+^ cells, IBA1^+^ microglia, or GFAP^+^ astrocytes indicated that loss of Sec13 did not alter their formation or recruitment (Supplemental Figure 9F). Ultrastructural analysis by electron microscopy further indicated that there were far fewer myelinated axons in the lesions of *Sec13*-iKO mice than in control mice (Figure 5J). The proportions of myelinating axons and the thickness of myelin sheaths, indicated as a g-ratio, were significantly decreased in *Sec13*-iKO mice (Figure 5, K and L). Notably, focal injection of lentivirus expressing Sec13 could restore the remyelination in *Sec13*-iKO mice (Supplemental Figure 9, G and H). Thus, these data suggest that Sec13 is crucial for the OL remyelination process following white matter injury.

### Ablation of Sec13 does not affect nucleocytoplasmic transport or mTOR signaling.

Sec13 is a multifunctional protein. In addition to its major function in the COPII complex, Sec13 also plays a role in the nuclear pore complex (NPC) (30). Therefore, we decided to determine whether the hypomyelination phenotype was caused by defects in nuclear transport. Staining with mAb414, a diagnostic NPC assembly marker that detects a subset of FG-Nups (31), showed similar staining intensity and structure between control and mutant Olig2^+^ OLs from spinal cord or primary cells (Supplemental Figure 10, A and B). Moreover, the levels of several nucleoporins were similar between control and mutant spinal cord protein extracts, indicating that the absence of Sec13 did not appear to change the proportions of other nucleoporins (Supplemental Figure 10C). Electron microscopy showed that optic nerves from *Sec13*-cKO mice also had an absence of nuclear envelope alteration or NPC clustering (Supplemental Figure 10D). As NPCs are critical for regulating the passage of molecules between the nucleus and the cytoplasm, we therefore investigated whether loss of Sec13 would affect general nucleocytoplasmic transport. OL cell line Oli-neu cells were transfected with tdTomato containing a nuclear localization signal (NLS) or nuclear export signal (NES) ( 31), and we observed no significant difference in the localization of tdTomato signals (Supplemental Figure 10E). To assess whether Sec13 depletion affects the transport of mRNAs, we performed oligo-dT ISH (32). We found no significant difference in the intracellular distribution of poly (A)^+^ RNA between control and Sec13-knockdown Oli-neu cells (Supplemental Figure 10F). Collectively, these data suggest that depletion of Sec13 does not affect global NPC assembly or function in OLs.

In addition, Sec13 also belongs to the GATOR2 complex, a positive regulator of the mTORC1 pathway (33). We thus tested the phosphorylation state of the S6 protein downstream of mTOR signaling. Knockdown of Sec13 in Oli-neu cells did not significantly inhibit the amino acid–induced activation of mTORC1, as detected by the phosphorylation state of S6 (Supplemental Figure 10, G and H). Thus, Sec13 may not regulate OL differentiation through major changes in mTOR signaling. Overall, these results suggest that Sec13 does not regulate OL differentiation through major alterations in nucleocytoplasmic transport or mTOR signaling.

### Sec13 regulates the OL secretome.

**Figure 6:**
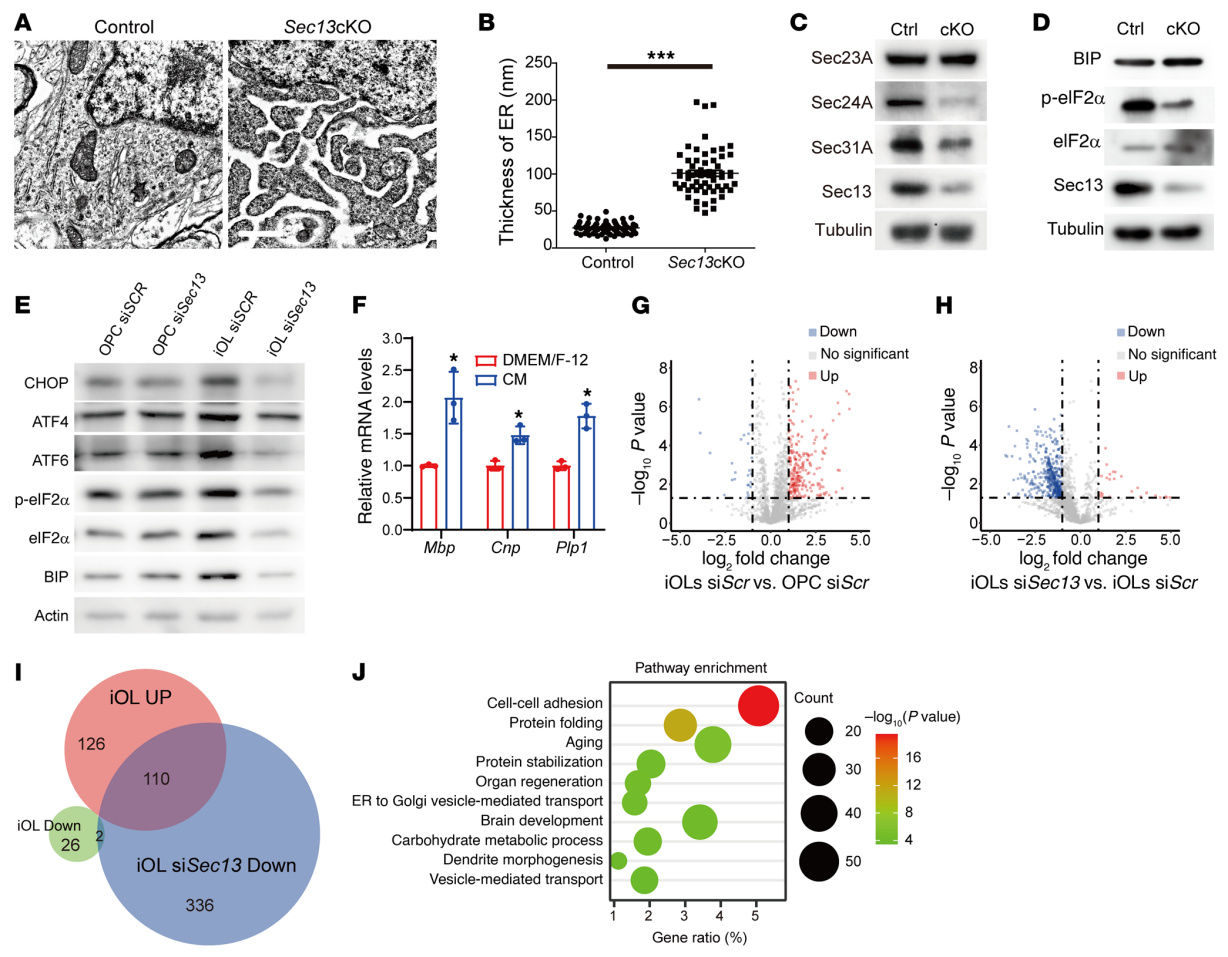
Sec13 regulates the OL secretome. (**A**) Electron microscopic analysis of the ER structure in spinal cords from control and *Sec13*-cKO mice at P7. Scale bars: 1 μm. (**B**) Quantification of ER thickness in spinal cords from control and *Sec13*-cKO mice at P7 (*n =* 3 independent experiments). (**C** and **D**) Immunoblotting for the indicated proteins in primary control and *Sec13*-cKO OPCs. (**E**) Immunoblotting for the indicated proteins in primary rat OPCs or differentiating immature OLs following treatment with scrambled (siSCR) or *Sec13* siRNAs. (**F**) Real-time PCR analysis of myelination-associated genes in primary rat OPCs under differentiation conditions following treatment with DMEM/F-12 or CM (*n =* 3 independent experiments). (**G**) Volcano plot showing the differential protein abundance (highlighted in color; fold change >2; *P* < 0.05) in the CM of primary rat differentiating immature OLs relative to that in OPCs. (**H**) Volcano plot showing the differential protein abundance (highlighted in color; fold change >2; *P* < 0.05) in the CM of primary rat differentiating immature OLs treated with *Sec13* siRNA relative to scrambled siRNA. (**I**) Venn diagram showing the overlap between differentially secreted factors upon OPC differentiation and knockdown of Sec13 in differentiating immature OLs. (**J**) GO functional category analysis of the proteins with reduced secretion after knockdown of Sec13. Data represent the mean ± SD. *P < 0.05 and ***P < 0.001, by 2-tailed, unpaired Student’s *t* test. Down, downregulated; Up, upregulated.

Sec13 is an essential component of COPII vesicles and is involved in protein trafficking from the ER to the Golgi organelle (24, 25, 34). Loss of COPII components has been reported to cause ER expansion due to intracellular protein accumulation (8, 34, 35). To explore the cause of the arrested OL differentiation in *Sec13*-cKO mice, we performed transmission electron microscopic analysis, which revealed that ablation of Sec13 caused ER distension in spinal cord from mutant mice (Figure 6, A and B). Meanwhile, we further found a concomitant decrease in the expression of Sec24A and Sec31A in mouse OPCs isolated from *Sec13*-cKO mice (Figure 6C), which is consistent with previous results with HeLa cells (36). The significant downregulation of Sec31A was confirmed by mouse primary OPC immunostaining results (Supplemental Figure 11A). Interestingly, we observed a compensatory upregulation of RNA levels in a subset of COPII components (Supplemental Figure 11B). Retention of excessive amounts of protein in the ER can lead to activation of the unfolded protein response (UPR) pathway (12). We therefore examined the expression of markers associated with UPR activation. Unexpectedly, the levels of phosphorylated eIF2α (p-eIF2α), a substrate of PKR-like ER kinase (PERK) (37), was not significantly increased by depletion of Sec13 (Figure 6D). Similarly, the levels of BIP, activating transcription factor 4 (ATF4), ATF6, and C/EBP homologous protein (CHOP), a transcription factor that promotes ER stress–mediated apoptosis (38), also remained unchanged in rat OPCs and spinal cord lysates after ablation of Sec13 (Figure 6E and Supplemental Figure 11C). Soluble proteins are packaged into the COPII complex and delivered to the Golgi organelle for further processing. In addition, several COPII components have been shown to regulate cell differentiation, chondrogenesis, and tumor metastasis by mediating the secretion of relevant proteins (7, 8, 11). We thus investigated whether proteins secreted by OLs would autonomously promote OL differentiation and whether loss of Sec13 impairs the secretion of these proteins. First, we assessed the ability of the conditioned medium (CM) from differentiating immature OLs to promote OL differentiation. Compared with plain DMEM/F-12 medium, CM significantly increased myelin gene transcription, indicating that differentiating immature OLs may regulate self-differentiation by autocrine signaling (Figure 6F). In contrast, CM from microglia or astrocytes showed a much weaker ability to affect the transcription of myelin genes (Supplemental Figure 11D). To better understand the underlying mechanism by which Sec13 regulates OL differentiation and to decipher the potential autocrine factors, we compared the secretome of OPCs and differentiating immature OLs in the absence or presence of an siRNA against Sec13. We detected a global increase in the secretion of proteins upon differentiation (Figure 6G), whereas knockdown of Sec13 strongly inhibited protein secretion (Figure 6H and Supplemental Table 1). We found that 47% (110 of 236) of the proteins that were originally upregulated in the secretome upon differentiation showed less abundance in CM from *Sec13*-knockdown, differentiating immature OLs (Figure 6I). Gene Ontology (GO) analysis revealed that proteins involved in cell adhesion and protein folding were most significantly affected by knockdown of Sec13 (Figure 6J). Together, these data suggest that Sec13 regulates the secretome of OLs.

### PTN promotes OL differentiation via autocrine signaling.

**Figure 7:**
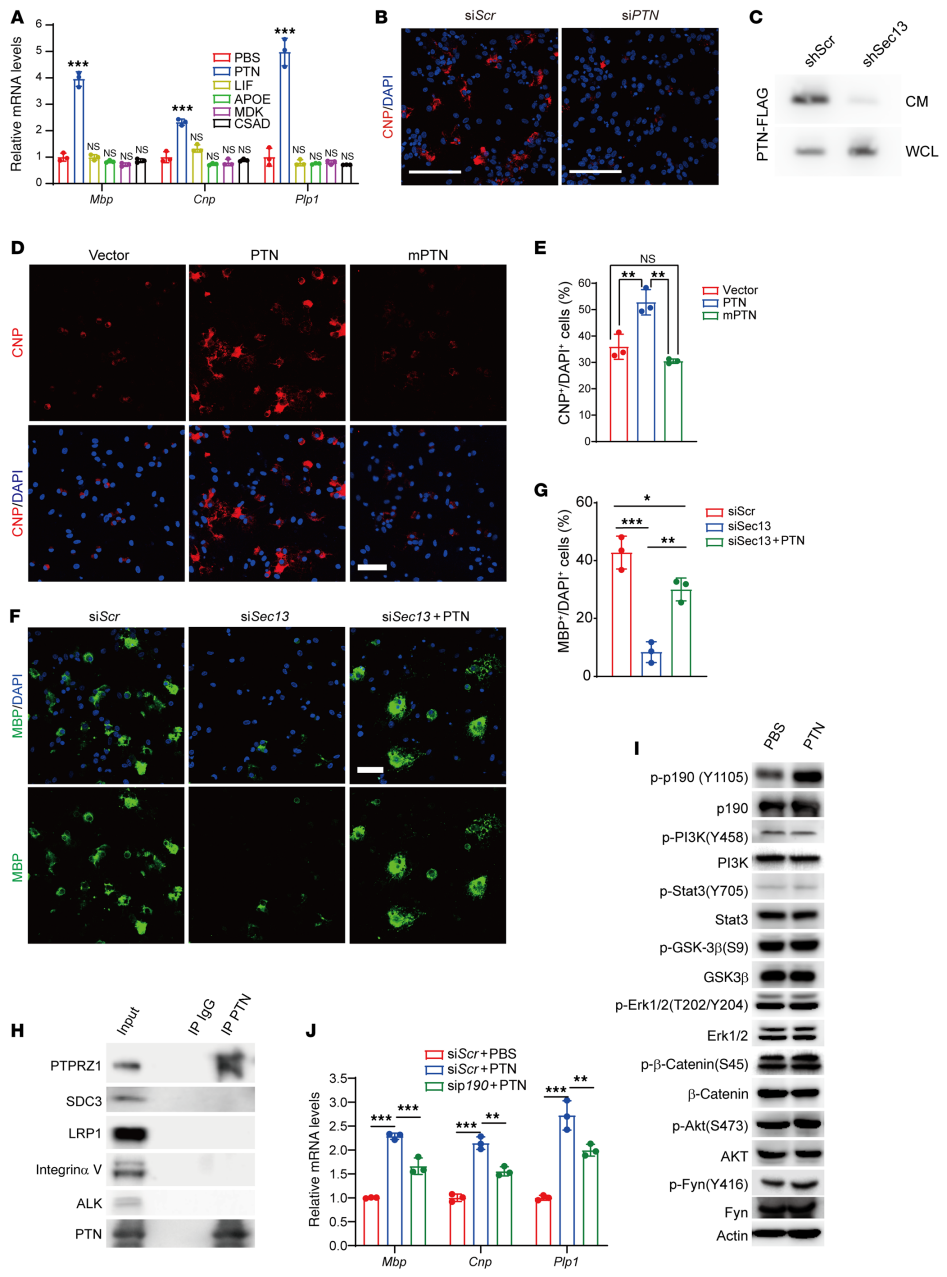
PTN promotes OL differentiation via autocrine signaling. (**A**) Real-time PCR analysis of myelination-associated genes in primary rat OPCs under differentiation conditions without T3, but in the presence or absence of the indicated recombinant factors (PTN, 100 nM; LIF, 50 ng/μL; APOE, 10 μg/μL; MDK, 100 nM; CSAD, 1.3 ng/μL). *n =* 3 independent experiments. (**B**) Immunolabeling for CNP in primary rat OPCs under differentiation conditions for 72 hours following treatment with scrambled or *PTN* siRNAs. Scale bars: 100 μm. (**C**) Immunoblotting for transfected PTN-FLAG in CM and cellular whole-cell lysate (WCL) from Oli-neu cells transduced with scrambled or *Sec13* shRNA. (**D** and **E**) Immunostaining (**D**) and quantification (**E**) of CNP signals in primary rat OPCs under differentiation conditions following PTN or PTN-mutant (L18&20R) overexpression (*n =* 3 independent experiments). Scale bar: 50 μm. (**F** and **G**) Immunostaining (**F**) and quantification (**G**) of MBP signals in primary rat OPCs under differentiation conditions following treatment with scrambled or *Sec13* siRNAs and recombinant PTN protein, respectively (*n =* 3 independent experiments). Scale bar: 50 μm. (**H**) Co-IP of the indicated endogenous factors with PTN in primary rat differentiating immature OLs. (**I**) Immunoblotting for the indicated proteins in primary rat differentiating immature OLs following treatment with recombinant PTN for 1 hour. (**J**) Real-time PCR analysis of myelination-associated genes in primary rat OPCs under differentiation conditions following treatment with scrambled or *p190* siRNAs and recombinant PTN protein, respectively (*n =* 3 independent experiments). Data represent the mean ± SD. *P < 0.05, **P < 0.01, and ***P < 0.001, by 1-way ANOVA with Tukey’s correction for multiple comparisons.

To uncover the central mediators of the secretome that promote OL differentiation, we next focused on those factors that have a reduced abundance after knockdown of Sec13. We functionally tested 5 candidates (PTN, leukemia inhibitory factor [LIF], apolipoprotein E [APOE], midkine [MDK], and cysteine sulfinic acid decarboxylase [CSAD]) by adding recombinant factors. Among the candidates, the addition of PTN, but not other factors, strongly potentiated myelin gene transcription (Figure 7A). In addition, MBP and CNP were induced by PTN at the protein level (Supplemental Figure 12, A–C). Meanwhile, depletion of PTN with an anti-PTN antibody impaired the ability of the CM to promote myelin gene transcription, indicating that PTN is the central mediator in CM that promotes OL differentiation (Supplemental Figure 12D). Knockdown of PTN inhibited myelin gene transcription and CNP expression (Supplemental Figure 12, E and F, and Figure 7B), phenocopying *Sec13* knockdown. We found that PTN transcript levels were substantially induced upon OL differentiation (Supplemental Figure 12G). Furthermore, in the developing corpus callosum, PTN expression peaked at the P8 perinatal stage in the Olig2^+^ OL lineage, just prior to myelination onset (Supplemental Figure 12H). Moreover, upon OL differentiation, PTN concentrated to the ERES, as indicated by the COPII component Sec24A (Supplemental Figure 12I). Taken together, our data indicate that PTN is highly expressed in differentiating OLs in the OL lineage.

PTN transcript levels remained unchanged after knockdown of Sec13 (Supplemental Figure 12J), however, we observed intracellular accumulation and reduced secretion of PTN in Sec13-knockdown Oli-neu cell lines (Figure 7C). Furthermore, analysis of PTN cellular distribution by immunofluorescence also demonstrated substantial accumulation in the ER of mouse differentiating immature OLs from Sec13-mutant mice, as indicated by the ER marker protein disulfide isomerase (PDI) (Supplemental Figure 13A). BFA disrupts the Golgi apparatus and, consequently, the delivery of ER-derived COPII vesicles along the secretory pathway (9). Treatment with BFA in differentiating immature OLs led to intracellular PTN accumulation (Supplemental Figure 13, B and C). These data suggest that COPII component Sec13–mediated ER-Golgi transport plays a critical role in PTN secretion. To further validate that secreted PTN has a direct effect on OL differentiation, we used a PTN mutant (L18&20R), which had a mutated signal peptide and could not be secreted into the medium (Supplemental Figure 13D). Ectopic expression of PTN, but not the PTN mutant, not only stimulated OPC differentiation into CNP^+^ OLs (Figure 7, D and E), but also upregulated the transcription of myelin genes (Supplemental Figure 13E). Because depletion of Sec13 inhibited OL differentiation and PTN secretion, we hypothesized that the exogenous replenishment of PTN could rescue the developmental defects of OLs in Sec13-knockdown cells. By adding PTN to OPCs, we found that PTN significantly increased the number of MBP^+^ OLs in Sec13-knockdown cells, indicating that PTN mediated Sec13 function in OL differentiation (Figure 7, F and G). Taken together, these data suggest that Sec13 functions as a COPII component to regulate OL differentiation primarily through mediation of PTN secretion.

We next examined how PTN regulates OL differentiation. PTN has been reported to function through several putative receptors, such as protein tyrosine phosphatase receptor type Z1 (PTPRZ1), anaplastic lymphoma receptor tyrosine kinase (ALK), chondroitin sulfate proteoglycan 5 (CSPG5), and syndecan 3 (SDC3) (39, 40). By immunoprecipitation, we found that PTN specifically bound to PTPRZ1 in differentiating immature OLs (Figure 7H). PTPRZ1 is a tyrosine phosphatase and is inactivated after binding with PTN (41). Previous studies have shown that PTN/PTPRZ signaling can activate several downstream effectors (42). Here, we found that exogenous PTN significantly increased the phosphorylation of p190RhoGAP, but not other reported effectors in OLs (Figure 7I). p190RhoGAP, a GTPase-activating protein (GAP), is phosphorylated at Tyr1105 during OL differentiation, and this modification enhances GAP activity, thereby suppressing the Rho/ROCK pathway and resulting in the maturation of OLs and myelination (43–45). Moreover, knockdown of p190RhoGAP attenuated the effect of exogenous PTN treatment on myelin gene transcription (Figure 7J). Collectively, these data demonstrate that Sec13-mediated PTN autocrine signaling promoted cell differentiation through the PTPRZ/p190RhoGAP pathway.

### PTN accelerates remyelination after demyelinating injury.

**Figure 8:**
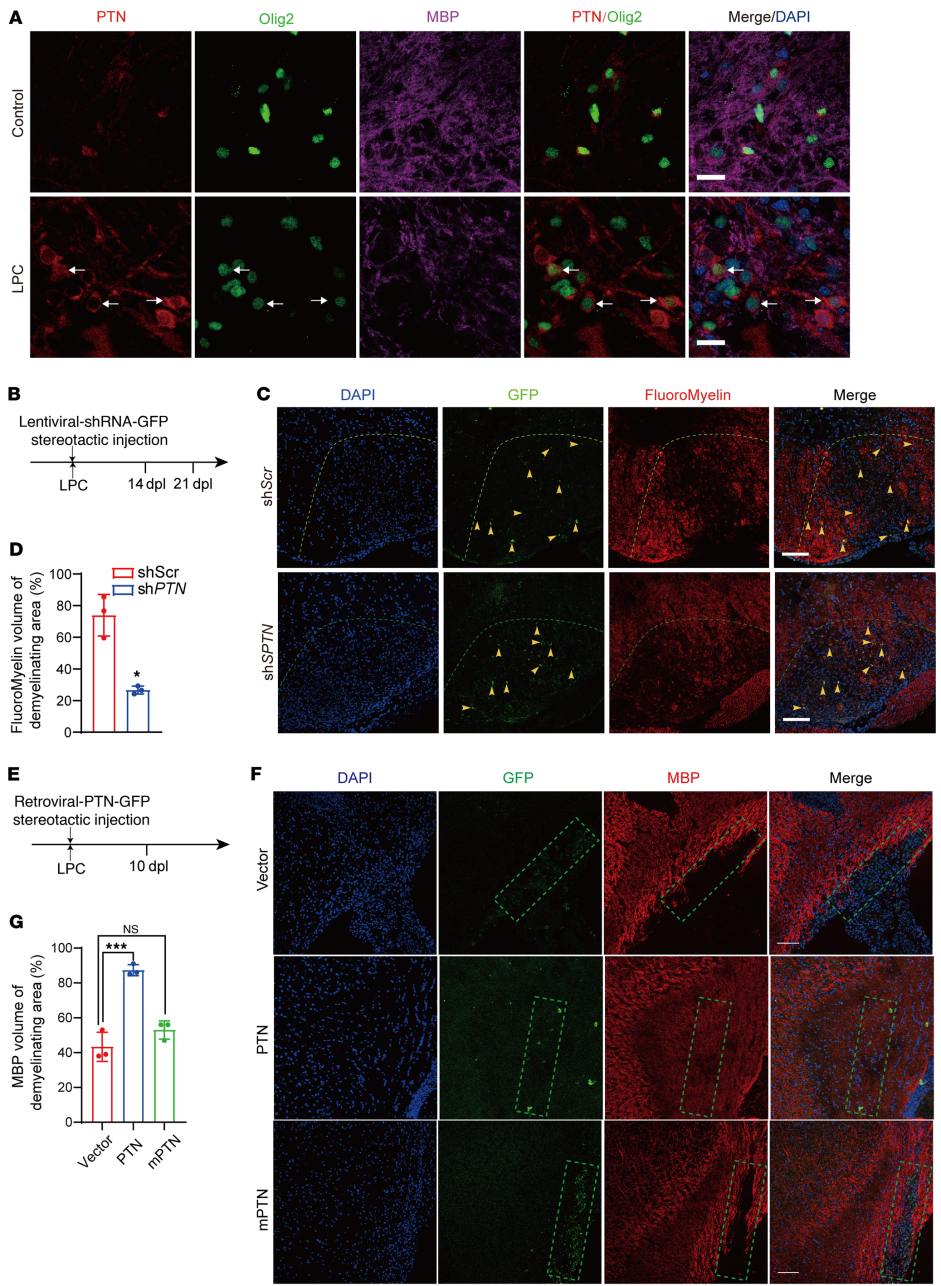
PTN accelerates remyelination after demyelinating injury. (**A**) Immunostaining for PTN, Olig2, and MBP at 7 dpl in spinal cord from nonlesion control and LPC-injured mice. Arrows indicate PTN^+^Olig2^+^ cells. Scale bars: 20 μm. (**B**) Diagram showing injection of LPC and lentivirus expressing shRNA against PTN into the spinal cord. (**C**) Representative images of GFP and FluoroMyelin in spinal cord lesions in mice after injection of lentivirus expressing scrambled or *PTN* shRNA at 14 dpl. Arrowheads indicate GFP^+^ cells. Scale bars: 100 μm. (**D**) Quantification of FluoroMyelin volume in spinal cord lesions of mice injected with lentivirus expressing scrambled or *PTN* shRNA at 14 dpl (*n =* 3 animals/treatment). Data represent the mean ± SD. *P < 0.05, by 2-tailed, unpaired Student’s *t* test. (**E**) Diagram showing injection of LPC and retrovirus expressing PTN or the PTN mutant (L18&20R) into the corpus callosum. (**F**) Representative images of GFP and MBP in corpus callosum lesions of mice after injection of retrovirus expressing PTN or the PTN mutant (mPTN, L18&20R) at 10 dpl. Scale bars: 100 μm. (**G**) Quantification of MBP volume in corpus callosum lesions of mice after injection of retrovirus expressing PTN or the PTN mutant (L18&20R) at 10 dpl (*n =* 3 animals/treatment). Data represent the mean ± SD. ***P < 0.001, by 1-way ANOVA with Tukey’s correction for multiple comparisons.

Given the PTN function in OL differentiation, we next asked whether PTN plays an important role in remyelination after demyelination injury. Although PTN was not previously detected in the OL lineage in the cuprizone-induced demyelinating model (46), we indeed observed that PTN was upregulated in Olig2-expressing cells upon LPC injury (Figure 8A). The discrepancy may result from the difference between LPC and cuprizone injury models, since LPC injection induces acute demyelination, unlike cuprizone treatment. By coinjecting lysolecithin lesions with lentivirus expressing shRNA against PTN into spinal cord (Figure 8B), we found that knockdown of PTN significantly impaired remyelination compared with the lentivirus expressing scrambled shRNA (Figure 8, C and D, and Supplemental Figure 14, A and B), whereas IBA1^+^ cell recruitment and lesion areas were comparable (Supplemental Figure 14, C–F). Similar results showing a decrease in remyelination were also obtained in the corpus callosum of mice injected with lentivirus expressing shRNA against PTN (Supplemental Figure 14, G–I). To further validate the role of PTN in remyelination, we next used a retrovirus expressing PTN or mutant PTN in the LPC demyelinating model (Figure 8E). On day 10 after injury, the expression of MBP and *Plp1* was significantly increased following administration of a retrovirus expressing PTN (RV-PTN) (Figure 8, F and G, and Supplemental Figure 15, A–C). By contrast, ectopic expression of the mutant PTN (L18&20R), which could not be secreted, did not substantially accelerate remyelination (Figure 8, F and G, and Supplemental Figure 15, A–C). The accelerated remyelination was confirmed by electron microscopic analysis of the corpus callosum, which revealed improved axonal ensheathment with an increased number of myelinated axons, as evidenced by ectopic expression of PTN (Supplemental Figure 15, D and E). These data demonstrate that PTN is required for remyelination and that secreted PTN is able to accelerate remyelination upon white matter injury.

## Discussion

### Physiological function of COPII in the CNS.

During OL differentiation, OLs undergo a 1000-fold increase in the membrane area and remarkable process extension (15), which is accompanied by a large amount of protein trafficking. Here, we investigated the expression patterns of COPII components during OL differentiation and the hypomyelination phenotype after specific depletion of Sec13 in the OL lineage. At the peak of myelination (P14) in the mouse CNS, COPII components were mainly found in OLs and neurons but were hardly detectable in astrocytes or microglia, which is conceivable, given the requirement of OLs and neurons for protein trafficking. Moreover, improving protein transport with TUDCA or inhibiting it with BFA can significantly affect OL differentiation. It is noteworthy that Sec13 and another COPII protein, Sec31A, were upregulated upon LPC-induced demyelination injury, indicating that protein trafficking was strongly required during myelin repair. Whether the expression levels of other COPII components will change in response to demyelination injury remains unanswered. The COPII complex captures cargo into vesicles and mediates vesicle budding from the ER. Although cargo recognition appears to be meditated primarily by the different isoforms of Sec24 (47), Sec23 and Sar1 also play a role in the recognition of a subset of cargos (48, 49). Here, we found that Sec23B, Sar1A, and Sar1B were also upregulated upon OPC differentiation. Our finding raises the question of whether specific upregulation of these COPII components will affect protein sorting or the secretome of OLs.

### Autocrine PTN signaling functions downstream of Sec13 in OLs.

By analysis of the secretome of OPCs and differentiating immature OLs, we showed that OLs secreted higher levels of factors upon differentiation induction, whereas knockdown of Sec13 strongly inhibited these secretions. We further identified PTN as an autocrine factor that promotes OL development. Instead of affecting PTN transcription levels, loss of Sec13 impaired PTN secretion and caused PTN accumulation in the ER. We also observed that PTN concentrated to the ERES upon differentiation. Through addition of recombinant PTN protein and ectopic PTN expression, we provide evidence that OLs were able to promote their own differentiation via autocrine PTN. OLs secreted high levels of factors according to the secretome we identified. The secretome was also altered upon differentiation. It would be intriguing to characterize the functions of the secreted factors under different developmental states. Compared with paracrine factors released by neurons or astrocytes, autocrine regulation has the advantage of spatial proximity of signals. It is reported that hypoxia or HIF1α stabilization activates cell-autonomous Wnt production and inhibits OPC maturation (50). Our finding provides another example that OPCs regulate their own differentiation by autocrine signaling and also highlights the complexity of regulatory mechanisms of myelinogenesis.

### Potential roles for PTN in remyelination.

PTN is a developmentally regulated factor that has diverse roles in brain development (39). It has been shown to function through several transmembrane receptors, such as PTPRZ, ALK, SDC3, and LRP1. By IP analysis, we found that only PTPRZ interacted with endogenous PTN in differentiating immature OLs. PTPRZ is an abundant phosphatase in OPCs that negatively regulates OPC maturation through its phosphatase activity (46). Furthermore, p190RhoGAP, which is a downstream dephosphorylating substrate of PTPRZ, can be quickly activated by PTN and induce OL process extension. In this study, we observed a dramatic upregulation of PTN in the OL lineage upon LPC-induced demyelinating injury, indicating a potential role of PTN in remyelination. More important, ectopic expression of PTN by a retrovirus accelerated the rate of remyelination in a mouse model of LPC-induced demyelination. Further studies are needed to determine whether infusion of PTN or a bioactive mimic is able to promote remyelination in other demyelinating models and human demyelinating diseases.

## Methods

### Mice.

*Sec13^fl/fl^* mice were generated by crossing hypomorphic Sec13 mice with FLPe-expressing transgenic mice to remove the NEO cassette (27). Mice homozygous for floxed alleles of *Sec13^fl/fl^* were crossed with *Olig1-Cre^+/–^* mice to generate *Sec13*-cKO (*Sec13 ^fl/fl^* *Olig1-Cre^+/–^*) and heterozygous control (*Sec13 ^fl/+^* *Olig1-Cre*^+/–^) mice (28). Heterozygous mice were used as controls, since they developed and behaved the same as WT mice. *NG2-CreERT* mice (51) or PDGFRα-CreERT (19) mice were crossed with *Sec13^fl/fl^* mice using a similar mating strategy to generate the OPC-specific iKO mice. *Sec13^fl/fl^* mice receiving tamoxifen were used as a control. Animals were housed under a 12-hour light/12-hour dark cycle with free access to water and food. All mice in the study were backcrossed on a C57BL/6 background for at least 6 generations.

### Antibodies and reagents.

The following antibodies were used: goat anti-MBP (catalog sc-13914; Santa Cruz Biotechnology); mouse anti-CC1 (catalog op-80; MilliporeSigma); rabbit anti-PDGFRα (catalog sc-338; Santa Cruz Biotechnology); rabbit anti-GFAP (catalog AP0123; Ascend); mouse anti-mAb414 (catalog 902901; BioLegend); goat anti-Olig2 (catalog AF2418; R&D Systems); mouse anti-NeuN (catalog MAB377; MilliporeSigma); rabbit anti-CNP (catalog 13427-1-AP; Proteintech); rat anti-BrdU (catalog ab6326; Abcam); rat anti-Ki67 (catalog 14-5698-82; Thermo Fisher Scientific); rabbit anti-Sec13 (catalog A303-980A; Bethyl Laboratories); mouse anti-Sec13 (catalog sc-7392; Santa Cruz Biotechnology); mouse anti-CNPase (catalog C5922; MilliporeSigma); rabbit anti-Olig2 (catalog AP0337; Talent Biomedical); rabbit anti-HA (catalog 51064-2-AP; Proteintech); rabbit anti-MBP (catalog BA0094; BOSTER); mouse anti-PTN (catalog sc-74443; Santa Cruz Biotechnology); rabbit anti-Flag (catalog 20543-1-AP; Proteintech); rabbit anti-Sec23A (catalog 8162; Cell Signaling Technology); rabbit anti-Sec23B (catalog ab151258; Abcam); anti-Sec24A (catalog 15958-1-AP; Proteintech); rabbit anti-Sec24B (catalog ab240703; Abcam); rabbit anti-Sar1A (catalog 15350-1-AP; Proteintech); rabbit anti-Sar1B (catalog 22292-1-AP; Proteintech); rabbit anti-Nup107 (catalog 19217-1-AP; Proteintech); mouse anti-tubulin (catalog 66031-1-Ig; Proteintech); mouse anti-Sec31A (catalog 612351; BD Biosciences); rabbit anti-Nup62 (catalog 13916-1-AP; Proteintech); mouse anti-GAPDH (catalog 60004-1-Ig; Proteintech); rabbit anti-Nup88 (catalog 55465-1-AP; Proteintech); rabbit anti-Nup205 (catalog 24439-1-AP; Proteintech), mouse anti–β-actin (catalog CL594-66009; Proteintech); mouse anti-Bip (catalog 66574-1-Ig; Proteintech); rabbit anti–p-eIF2a (catalog ab32157; Abcam); rabbit anti-eIF2a (catalog 11233-1-AP; Proteintech); rabbit anti-CHOP (catalog 15204-1-AP; Proteintech); mouse anti-Atf4 (catalog CL594-60035; Proteintech); rabbit anti-Atf6 (catalog 24169-1-AP; Proteintech); rabbit Anti-PDI (catalog 11245-1-AP; Proteintech); rabbit anti-PTPRZ1 (catalog 55125-1-AP; Proteintech); rabbit anti-SDC3 (catalog A18312; ABclonal); rabbit anti-LRP1 (catalog A0633; ABclonal); rabbit anti–integrin α V (catalog A19071; ABclonal); rabbit anti-ALK (catalog A0766; ABclonal); rabbit anti–p-p190 Y1105 (catalog P30433; BOSTER); rabbit anti-p190 (catalog 26789-1-AP; Proteintech); rabbit anti–p-PI3K (catalog 4228; Cell Signaling Technology); rabbit anti-PI3K (catalog 3358; Cell Signaling Technology); mouse anti–p-Stat3 Y705 (catalog 4113; Cell Signaling Technology); mouse anti-Stat3 (catalog 9132; Cell Signaling Technology); rabbit anti–p-GSK-3β (S9) (catalog 9336; Cell Signaling Technology); rabbit anti-GSK3β (catalog 9332; Cell Signaling Technology); rabbit anti–p-ERK1/2 (T202/Y204) (catalog 4376; Cell Signaling Technology); rabbit anti-ERK1/2 (catalog 9102; Cell Signaling Technology); rabbit anti–p-β-Catenin (S45) (catalog 9564; Cell Signaling Technology); mouse anti–β-catenin (catalog c19220; Cell Signaling Technology); rabbit anti–p-AKT (S473) (catalog 9271; Cell Signaling Technology); mouse anti-AKT (catalog CL488-60203; Proteintech); rabbit anti–p-Fyn (Y416) (catalog 2102; Cell Signaling Technology); mouse anti–ribosomal proteins s6 (catalog sc-74576; Santa Cruz Biotechnology); rabbit anti–p–S6 ribosomal protein (catalog 2211; Cell Signaling Technology); rabbit anti-FYN (catalog CL488-66606; Proteintech); Cy2 AffiniPure Donkey Anti–Mouse IgG (H+L) (catalog 715-225-151; Jackson ImmunoResearch Laboratories); Cy3 AffiniPure Donkey Anti–Rabbit IgG (H+L) (catalog 711-165-152; Jackson ImmunoResearch Laboratories); and Cy5 AffiniPure Donkey Anti–Goat IgG (H+L) (catalog 705-175-147; Jackson ImmunoResearch Laboratories). The following reagents were used: T3 RNA polymerase (catalog P208C; Promega); DIG RNA label mix (catalog 11277073910; Roche); LPC (catalog L4129; MilliporeSigma); tamoxifen (catalog T5648; MilliporeSigma); Prolong Gold Antifade Mountant (catalog P36934; Thermo Fisher Scientific); PTN (100 nM, catalog 51000; Sino Biological); PDGF-AA (catalog 100-13A; Peprotech); basic FGF (bFGF) (catalog 10014-HNAE; Sino Biological); NT3 (catalog 450-03; Peprotech); LIF (50 ng/mL, catalog 50756; Sino Biological); APOE (15 μg/mL, catalog 10817-H30E; Sino Biological); MDK (100 nM, catalog 10247; Sino Biological); CSAD (250 ng/mL, catalogH00051380-Q01; NOVUS); FluoroMyelin (catalog F34652; Thermo Fisher Scientific); BFA (catalog 50502ES03; Yeason Biotech); CNTF (catalog 450-13; Peprotech); and a TUNEL kit (catalog A113-03; Vazyme).

### Western blotting.

Proteins from each sample were separated on 10%–15% SDS-PAGE gels and transferred onto PVDF membranes. The membranes were blocked with 5% nonfat milk powder in TBS containing 0.1% Tween-20 for 1 hour, followed by incubation with primary antibodies at 4°C overnight. After washing, the membranes were incubated with HRP-conjugated secondary antibodies and developed using an ECL detection system (Sangon Biotech, catalog C510043) according to the instructions of the manufacturer.

### Cell culturing and analyses.

Primary mouse OPCs were isolated as described previously (52). Briefly, P4–P7 mouse cortices cells were obtained by sequential immunopanning with antibodies against GalC and anti-O4 antibody–coated plates. Rat OPCs were isolated from cortices of pups at P2–P4, as with mouse OPCs, with slight modifications. Briefly, mixed cortical cells were obtained by sequential immunopanning with antibodies against GalC and anti-A2B5 antibody–coated plates. Isolated OPCs were grown in OPC growth medium (OGM) (DMEM/F-12, Gibco, Thermo Fisher Scientific) with the addition of 2% B-27 (Gibco, Thermo Fisher Scientific); 1% N2 (Gibco, Thermo Fisher Scientific); 20 ng/mL PDGF-AA (Peprotech, 100-13A); 10 ng/mL CNTF (Peprotech, 450-13); 20 ng/mL bFGF (Sino Biological, 10014-HNAE); 5 μΜ forskolin (MilliporeSigma, F6886); 10 ng/mL biotin (MilliporeSigma, B4639); 5 μg/mL insulin (MilliporeSigma, I-6634); trace elements B (Corning, 16615008); sodium pyruvate (Invitrogen, Thermo Fisher Scientific, 11360-070); penicillin-streptomycin (Invitrogen, Thermo Fisher Scientific, 15140-122); and 1 ng/mL NT3 (Peprotech, 450-03). OPCs were differentiated using OPC differentiation medium (ODM). This medium differed from the OGM, in that T3 (60 nM) was added, and PDGF-AA, bFGF, and NT3 were removed.

Primary microglial cultures were obtained by microdissection of brains of neonatal mice (<2 days old). The brains were mechanically minced and dissociated with 0.25% trypsin., and the tissue suspension was passed through a 70 μm nylon cell strainer. Cell pellets were harvested and resuspended in DMEM supplemented with 10% heat-inactivated FBS and plated onto poly-l-lysine–precoated culture flasks. After 3 days, the medium, containing 25 ng/mL GM-CSF and 10% FBS was changed. Primary microglial cells were harvested by shaking (200 rpm, for 20 minutes) after 10–12 days in culture and every 3 days thereafter. Primary astrocytes were cultured in astroglial medium (DMEM/F12, 1:1) (Gibco, Thermo Fisher Scientific) with 10% FBS. Contaminating cells in the astroglia monolayer were removed by overnight shaking at 220 rpm at 37°C.

Oli-neu cells (53) were cultured in Oli-neu medium, which contained DMEM/F-12 supplemented with 2% B-27, 1% N2, 5% FBS (Gibco, Thermo Fisher Scientific), 1% horse serum (Gibco, Thermo Fisher Scientific), 7.2 mM glucose, and 1× penicillin-streptomycin. The 293T cells (obtained from the American Type Culture Collection [ATCC]) were maintained in DMEM supplemented with 10% FBS and 1× penicillin-streptomycin. Sinofection (Sino Biological, STF02) was used as a transfection reagent for the 293T cells. Electroporation was performed in a cuvette provided with the Nucleofector Kit using a Lonza Nucleofector 2b device (Lonza, O-017) according to the manufacturer’s protocol. Approximately 2.0 × 10^6^ OPCs or Oli-neu cells in 100 μL Basic Nucleofector Kit for Primary Mammalian Glial Cells (Lonza, VPI-1006) were resuspended. Five micrograms of plasmids or 5 μL of 20 μM siRNA was used for each transfection. Generally, the cells were kept in growth medium for 24 hours after electroporation and then incubated in differentiating medium for 48 or 72 hours.

### RNA extraction and quantitative real-time PCR.

Total RNA was extracted according to the TRIzol (Life Technologies, Thermo Fisher Scientific) protocol, and cDNA was produced with 5× All-In-One RT MasterMix (Applied Biological Materials [abm]). The following primer sequences were used to identify (a) the allele containing the NEO cassette: *Neo* forward, ATGTGTCAGTTTCATAGCCTGAAG, *Neo* reverse, CAGGTGAGGGTTCCAAGACC; and (b) the allele deleted from the NEO cassette: *dNeo* forward, GCTAATAAAATCATATTGCA, *dNeo* reverse, CAGGTGAGGGTTCCAAGACC. Real-time PCR was carried out using the Bio-Rad Real-Time PCR System with ChamQ Universal SYBR qPCR Master Mix (Vazyme). The following primer sequences were used for the rat genes: *Gapdh* forward, TCCAGTATGACTCTACCCACG, *Gapdh* reverse, CACGACATACTCAGCACCAG; *Cnp* forward, CTACTTTGGCAAGAGACCTCC, *Cnp* reverse, AGAGATGGACAGTTTGAAGGC; *Plp* forward, TCTTTGGCGACTACAAGACCACCA, *Plp* reverse, CAAACAATGACACACCCGCTCCAA; *Mbp* forward, TTGACTCCATCGGGCGCTTCTTTA, *Mbp* reverse, TTCATCTTGGGTCCTCTGCGACTT; *Sec13* forward, GGTCACCTAAACTCCTACACAAG, *Sec13* reverse, CATCCACCGACTCTTTCCAC; *Sec23A* forward, GAGCAAAACTCTGGGCTTGC, *Sec23A* reverse, GGGACCACGCAGAACTACAT; *Sec23B* forward, AGAACGAGATGGTGTGCGTT, *Sec23B* reverse, GGTAAGTCTGGGCGCTCTTT; *Sec24A* forward, TCCCCGAATGGCACTACCTA, *Sec24A* reverse, GTCTGTGGTCCTGTGGATGG; *Sec24B* forward, CAGCAATTAACGAAAATGTCCAAC, *Sec24B* reverse, TGCCTTTTGTCTGCATCTGCT; *Sar1A* forward, GGCTCTATGGGCAAACCACA, *Sar1A* reverse, CCTTGCCTCTTGAGCACACT; *Sar1B* forward, CGTCCCAACACTACATCCCA, *Sar1B* reverse, TCCATACTCTTCGGGCTTGC; *Sec31A* forward, CAGCCAGCCACCACCTTATC, *Sec31A* reverse, AGAAGCAGGAGGAGCAACAG; *Sec31B* forward, TCACGGCCAAGTGAGAAGAC, *Sec31B* reverse, TCTTCAGGCATGTGTCCACC; *PTN* forward, GGCTTGGGGAGAATGTGACC, *PTN* reverse, ACAGGGCTTGGAGATGGTGA; *P190* forward, TAGCATCCGAAAGAGCCGGT, *P190* reverse, GCCATCAGTGAGTGCGACAA. Primer sequences for the mouse genes were: *Gapdh* forward, TGCCAAATATGATGACATCAAGAA, *Gapdh* reverse, GGAGTGGGTGTCGCTGTTG; *Sec13* forward, GACTGGGTCCGAGATGTTG, *Sec13* reverse, ACTTGTGTAGGAGTTTAGGTGAC; *Mbp* forward, TCACAGAAGAGACCCTCACA; *Mbp* reverse, GCCGTAGTGGGTAGTTCTTG; *Cnp1* forward, TCCACGAGTGCAAGACGCTATTCA, *Cnp1* reverse, TGTAAGCATCAGCGGACACCATCT; *Plp* forward, TGCTCGGCTGTACCTGTGTACATT, *Plp* reverse, TACATTCTGGCATCAGCGCAGAGA; *Mog* forward, AGATGGCCTGTTTGTGGAG, *Mog* reverse, TTCATCCCCAACTAAAGCCC; *Myrf* forward, CAGACCCAGGTGCTACAC, *Myrf* reverse, TCCTGCTTGATCATTCCGTTC.

### Tissue preparation and immunohistochemistry.

Mice were anesthetized before sacrifice. The brain and spinal cord were dissected, fixed with 2% paraformaldehyde (PFA) for 6–8 hours and dehydrated in 30% sucrose. Tissues were embedded in OCT compound (CellPath) and sliced into 12 μm sections using a cryostat. The cryosections were permeabilized and incubated with blocking solution (0.4% Triton X-100 and 3% normal BSA in PBS) for 1 hour at room temperature (RT) and overlaid with primary antibodies at various dilution ratios overnight at 4°C. The sections were incubated with secondary antibodies, which were conjugated to Cy2, Cy3, and Cy5 before mounting. Immunofluorescence images were obtained using a confocal laser microscope (Leica SP8, Zeiss LSM 880 Airyscan, or Zeiss LSM 780). For immunostaining, cells were first fixed by 2% PFA for 30 minutes at RT. Subsequently, cells were permeabilized with the 0.5% Triton X-100 in PBS at 4°C and then blocked by 3% BSA in PBS. The samples were incubated with primary antibodies overnight at 4°C. After washing in PBS, secondary antibodies were added and incubated at RT for 1 hour before mounting with ProLong Gold Antifade Reagent (Invitrogen, Thermo Fisher Scientific). For ISH, cryosections were incubated with the digoxigenin-labeled (DIG-labeled) RNA antisense probe for murine *Plp1*/*Dm-20* as described previously (54). The probes were prepared with a DIG RNA labeling kit (Roche, 11277073910). An anti-DIG antibody conjugated with alkaline phosphatase (Roche, 11093274910) was used to probe the sections. Nitro blue tetrazolium (NBT) and 5-bromo-4-cloro-3-indolyl phosphate (BCIP) (Sangon, A600116) were used for substrate development.

Oligo d(T) ISH was performed as described previously (32). Briefly, cells were fixed with PFA and permeabilized with Triton X-100. Then, the hybridization was performed at 42°C overnight with a cy3-labeled oligonucleotide (dT) probe. The samples were washed with 2 × and 0.5× SSC at 42°C, stained with DAPI, and then mounted onto glass slides.

### LPC-induced demyelination injury.

LPC-induced demyelination was carried out in the corpus callosum or spinal cord of 10-week-old mice as described previously (31). Briefly, 1 μL 1% LPC was injected with a Hamilton syringe into the ventrolateral white matter in the spinal cord between Th3 and Th4. For demyelination in the brain, demyelinating lesions were induced by microinjection of 1.5 μL 1% LPC into the corpus callosum at the following coordinates: 1.0 mm backward to bregma, 1.0 mm lateral to bregma, and 1.5 mm deep relative to the skull surface. Titers of lentivirus and retrovirus were estimated to be approximately 1.0 × 10^5^/μL, and 1 μL virus was injected by mixing with LPC in the indicated experiments. Mice were sacrificed at different time points after injury. For TUDCA treatment in the LPC injury assay, mice were treated with TUDCA (500 mg/kg) by gavage over 11 consecutive days. Ethidium bromide-induced (EB-induced) demyelination was carried out in a manner similar to the LPC injury assay. EB (1%, 1.5 μL) was microinjected into the same regions as in the LPC assay.

### Tamoxifen administration.

Tamoxifen (20 mg/mL) was prepared in corn oil and stored at –20°C. A dose of 40 μg/g (grams per body weight) was i.p. injected into newborn mice once a day for 3 days from P3 to P5. For the LPC assay, 8- to 10-week-old mice were treated with tamoxifen (200 μg/g) by gavage over 7 consecutive days.

### Plasmids and virus preparation.

Mouse *Sec13* (gene ID: 110379), rat *Sec13* (gene ID: 297522), rat *Sec31A* (gene ID: 93646), mouse *Ptn* (gene ID: 19242), and rat *Ptn* (gene ID: 24924) were cloned into the pcDNA3.3, RV-GFP, or pCDH-MSCV-T2A-copGFP vectors. The 18th and 20th amino acids were mutated from leucine (L) to arginine (R) to make the PTN-L18/20R plasmid. Lentiviruses were generated by transfecting 293T cells with the lentiviral vector pLKO.1 and packaging plasmids. Retroviruses were generated by transfecting 293T cells with the retroviral vector and packaging plasmids. Viruses were concentrated by ultracentrifugation.

### IP analysis.

Cells were washed twice with ice-cold PBS and scraped off with PBS supplemented with the protease inhibitor PMSF (1 mM). For each sample, approximately 1.0 × 10^7^ cells were lysed with 1 mL co-IP buffer (50 mM Tris-HCl, pH 7.5, 150 mM NaCl, 1 % Triton X-100, 1 mM EDTA, 0.0 1 mM NaF, 0.01 mM Na_3_VO_4_) with 1 mM PMSF for 1 hour. The samples were then briefly sheared by sonication 10 times (1 second on, 1 second off, power = 25%). After centrifugation at 4°C for 15 minutes at 14,000*g*, 5% of the supernatants were boiled with 4× SDS as an input, and the remaining supernatant was incubated with 2 μg antibody overnight. Immunoprecipitated complexes were collected using 30 μL of 50% slurry Protein A/G Plus Agarose Beads (MilliporeSigma) at 4°C for 2 hours. Beads were washed 3 times with co-IP buffer. After washing, the beads were resuspended in 30 μL 2× SDS and boiled at 95°C for 15 minutes for elution.

### CM.

Microglia (at 80% confluence), astrocytes, and rat immature OLs (differentiated for 48 hours) were washed 3 times with PBS and changed to DMEM/F-12 for 24 hours to collect secreted proteins. Collected DMEM/F-12 medium was centrifuged at 400*g* for 5 minutes and filtered with 0.45 μm filters to removed cell debris. Each 20 mL of DMEM/F-12 from these glia cells and 20 mL of fresh DMEM/F-12 were concentrated to 1.8 mL with an Amicon Ultra-15 Centrifugal Filter (Merck). For PTN depletion, concentrated medium was incubated with 2 μg PTN antibody overnight following incubation with 30 μL 50% slurry Protein A/G Plus Agarose Beads. The sample was centrifuged to remove PTN. 10× OPC Differentiation Medium components (0.2 mL, without T3) in DMEM/F-12 were added to the concentrated medium to make CM. Rat OPCs (1 × 10^6^) in a 35 mm dish were treated with CM for 48 hours.

### Electron microscopy.

Tissue and cell processing was performed essentially as described previously (31). Briefly, mice were anesthetized and perfused with pre-cooled sodium cacodylate buffer. Optic nerves, corpus callosum, and spinal cord were immediately dissected and immersed in a pre-cooled fixation buffer (2.5% glutaraldehyde, 0.1 M phosphate buffer [PB], pH 7.4) overnight at 4°C. OPCs were directly dissociated and collected as sedimentary bulks, which were then fixed as described above. After being washed in PB, the samples were successively treated with OsO_4_ (1% OsO_4_ in PB), serially dehydrated in ethanol, and finally embedded in Spurr’s resin to obtain the blocks. The blocks were then sectioned into 70 nm slices and stained with lead citrate for electron microscopic imaging using a Hitachi HT-7800.

### LC-MS/MS analysis of CM.

Each group contained 3 duplications. For each sample, rat OPCs (3 × 10^^6^ cells) were differentiated with ODM for 48 hours and then washed 3 times with PBS and changed to DMEM/F-12 for 3 hours to collect secreted proteins. The supernatants were mixed with a 20% volume of 100% w/v TCA and incubated at 4°C overnight. The protein pellet was collected by centrifugation at 21,000*g* for 15 minutes and washed with pre-cooled acetone, and finally followed by centrifugation at 21,000*g* for 10 minutes. The wash step was repeated twice. Then, the protein pellet was diluted with 1% sodium deoxycholate in 0.1 M Tris, pH 8.0, and the concentrations were determined using the BCA Protein Quantification Kit (Vazyme). Label-free protein quantification was performed using TripleTOF 5600 LC-MS/MS system (AB Sciex).

### Statistics.

Data are presented as the arithmetic mean ± SEM. Statistical analyses were performed with GraphPad Prism 6 (GraphPad Software), and significance was set at a *P* value of less than 0.05 using a 2-tailed, unpaired Student’s *t* test or 1-way ANOVA with Tukey’s correction for multiple comparisons. Each experiment was conducted by analyzing at least 3 different experimental groups in a blinded fashion.

### Study approval.

The care and treatment of animals were approved by the IACUC of Xiamen University.

## Author contributions

LZ, ZW, and WM conceived and designed the study and experiments. ZL, MY, WL, RJ, WD, JC, Chaomeng Wang, LL, MW, XL, Caiming Wu, CX, LY, Chaoying Wang, JH, and NX performed the experiments. ZL, MY, and LZ analyzed data. LZ wrote the manuscript and supervised the study. The order of co–first authors was determined by the volume of work each contributed to the study.

## Supplementary Material

Supplemental data

Supplemental table 1

## Version 1 02/10/2022

In-Press Preview

## Version 2 04/01/2022

Electronic publication
