# Supplementary material for: Sec13 promotes oligodendrocyte differentiation and myelin repair through autocrine pleiotrophin signaling
Source: J Clin Invest. 2022 Apr 1;132(7):e155096. doi: 10.1172/JCI155096 (PMC8970680; doi:10.1172/JCI155096)
Supplement: Supplemental data [file jci-132-155096-s076.pdf]

Supplemental Information

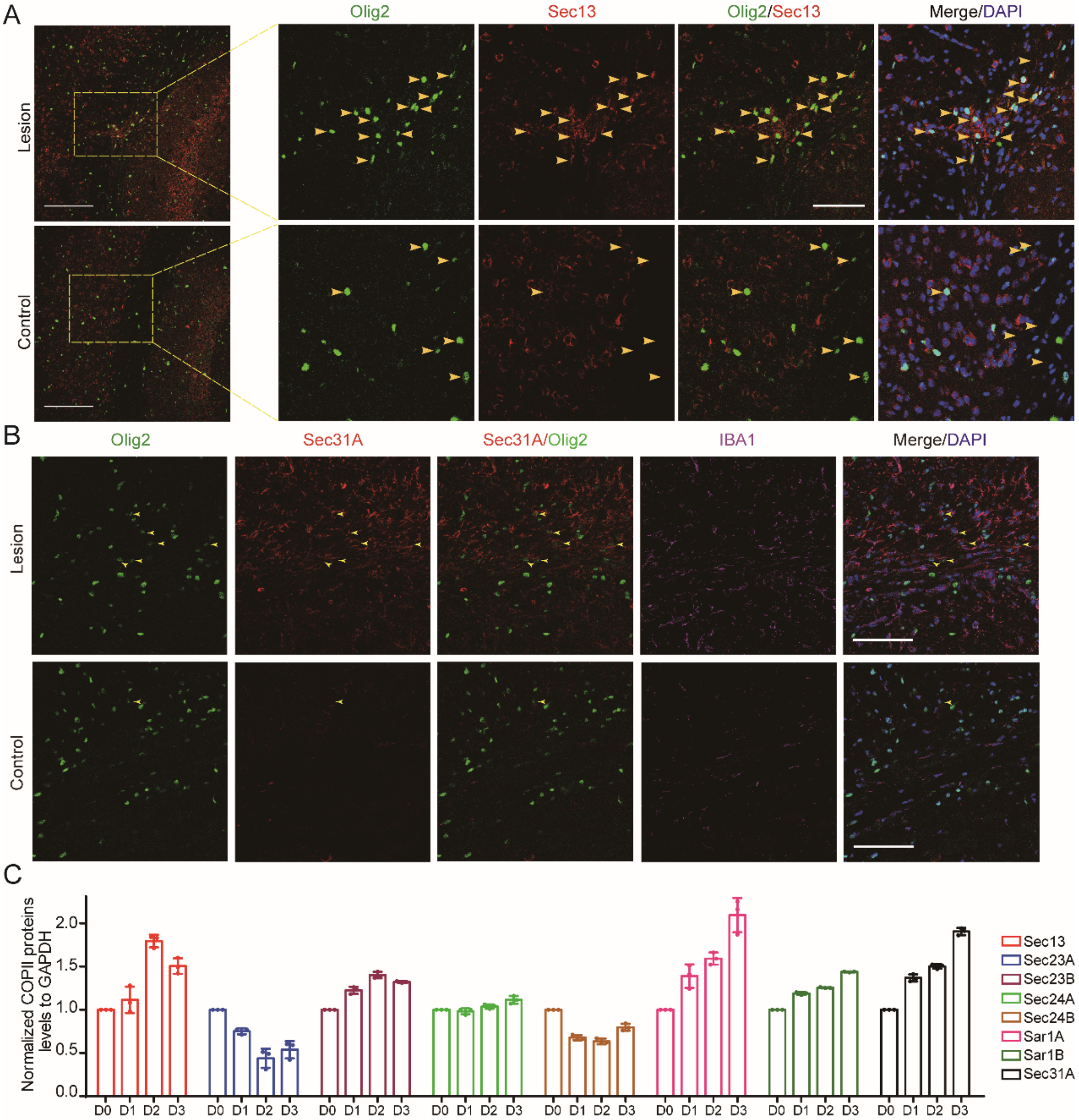

Supplemental Figure 1. COPII complex is implicated in remyelination after demyelination.

(A) Immunostaining of Olig2 and Sec13 at 7 dpl in corpus callosum from non-lesion control and EB lesion mice. Boxed area is shown on the right. Arrowheads indicate Olig2$^+$ cells. Scale bars represent 200 μm in left and 80 μm in right.

(B) Immunostaining of Olig2, Sec31A and IBA1 at 7 dpl in corpus callosum from non-lesion control and EB lesion mice. Arrowheads indicate Olig2$^+$ cells. Scale bars represent 100 μm.

(C) Histograms show fold changes of COPII components measured by densitometry after normalization with GAPDH (n= 3 independent experiments, D, day after differentiation).

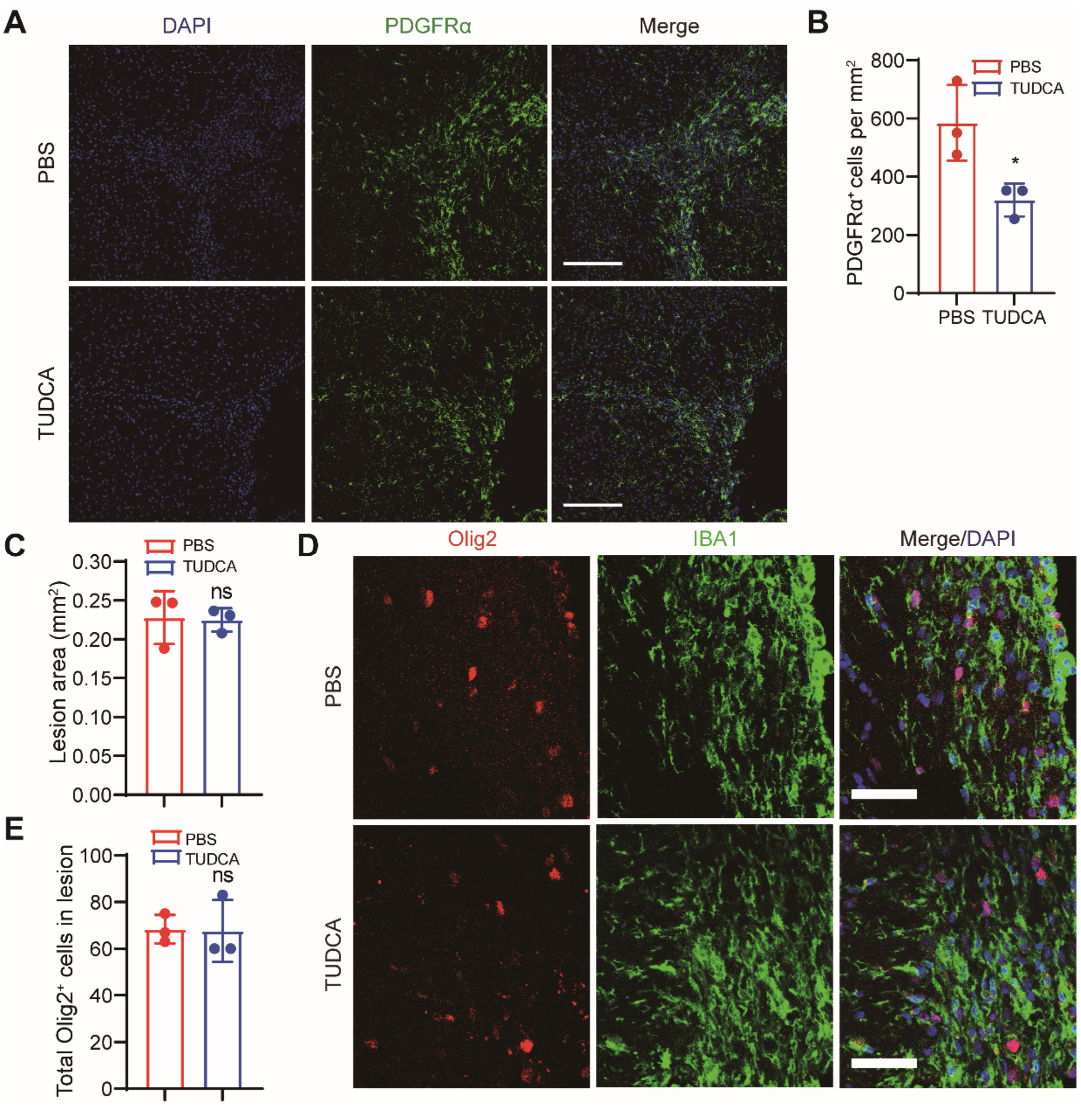

Supplemental Figure 2. The number of oligodendrocyte lineage cells appear unaltered after TUDCA treatment.

(A) Immunostaining of PDGFRα in corpus callosum lesions of control and TUDCA-treated mice at 10 dpl. Scale bars represent 200 μm.

(B) Quantification of PDGFRα^{+} cells in LPC lesion sites (n= 3 animals/treatment).

(C) Quantification of lesion area in LPC lesion sites of control and TUDCA-treated mice at 10 dpl (n= 3 animals/treatment).

(D) Immunostaining of Olig2 and IBA1 in corpus callosum lesions of control and TUDCA-treated mice at 10 dpl. Scale bars represent 50 μm.

(E) Quantification of Olig2^{+} cells in LPC lesion sites of control and TUDCA-treated mice at 10 dpl (n= 3 animals/treatment). Data are represented mean ± SD; *P<0.05, two-tailed unpaired Student's t test.

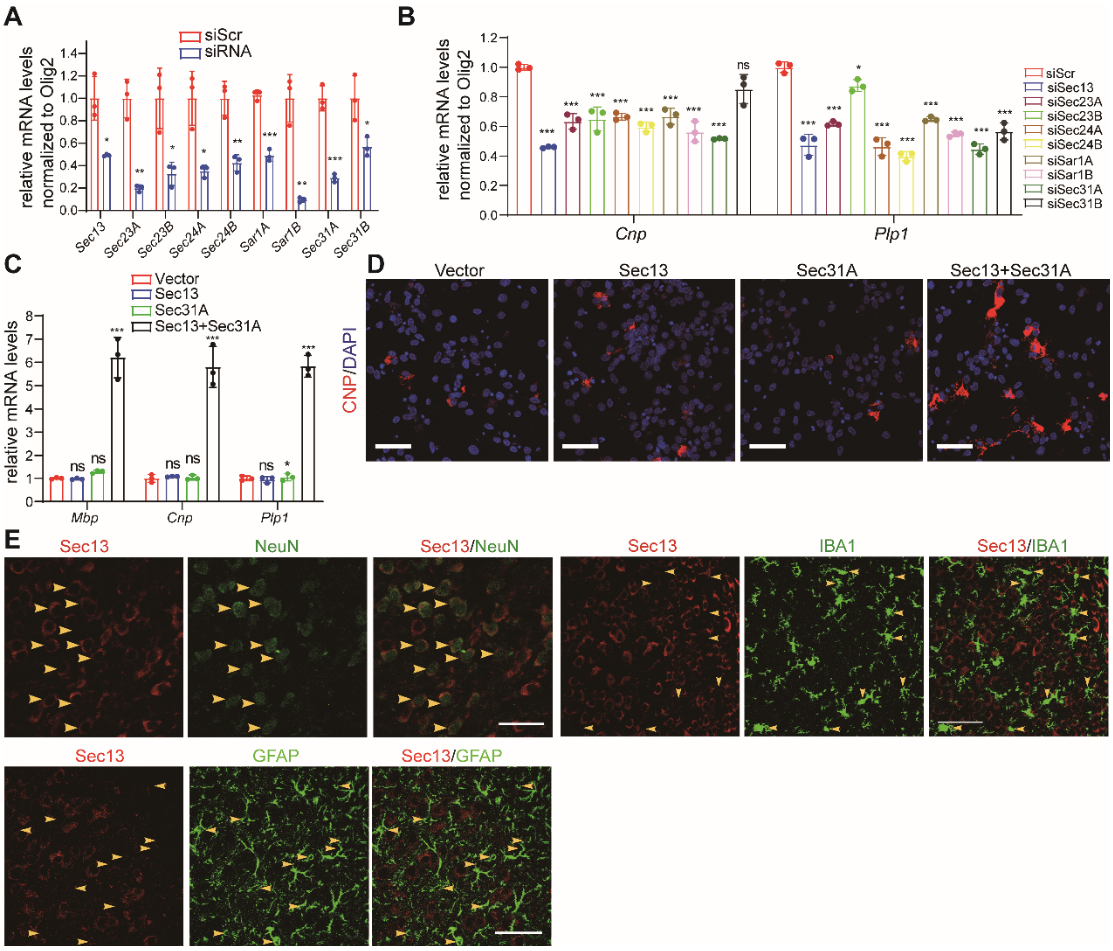

A
siScr
siRNA
relative mRNA levels normalized to Olig2
Sec13
Sec23A
Sec23B
Sec24A
Sec24B
Sar1A
Sar1B
Sec31A
Sec31B
B
relative mRNA levels normalized to Olig2
Cnp
Plp1
siScr
siSec13
siSec23A
siSec23B
siSec24A
siSec24B
siSar1A
siSar1B
siSec31A
siSec31B
C
Vector
Sec13
Sec31A
Sec13+Sec31A
relative mRNA levels
Mbp
Cnp
Plp1
D
Vector
Sec13
Sec31A
Sec13+Sec31A
CNP/DAPI
E
Sec13
NeuN
Sec13/NeuN
Sec13
IBA1
Sec13/IBA1
Sec13
GFAP
Sec13/GFAP

Supplemental Figure 3. COPII components are necessary for oligodendrocyte differentiation.

(A) Real-time PCR analysis of COPII components in primary rat OPCs following treatment with scrambled or indicated siRNAs, respectively (n=3 independent experiments). Data were analyzed by two-tailed unpaired Student's *t* test.

(B) Real-time PCR analysis of *Cnp* and *Plp1* expression in primary rat OPCs under differentiation conditions following treatments with indicated siRNAs (n=3 independent experiments). Data were analyzed by 1-way ANOVA with Tukey's correction for multiple comparisons.

(C-D) Real-time PCR analysis of myelination-associated genes (C) and immunostaining of CNP (D) in primary rat OPCs under differentiation conditions following transfection with Sec13 or Sec31A or Sec13 plus Sec31A construct (n=3 independent experiments). Scale bars represent 50 μm. Data were analyzed by 1-way ANOVA with Tukey's correction for multiple comparisons.

(E) Immunostaining of Sec13, neuron marker NeuN, astrocyte marker GFAP and microglia marker IBA1 in the cortex adjacent to corpus callosum (Sec13/NeuN) or corpus callosum (Sec13/GFAP/IBA1) of mice at P14. Scale bars represent 30 μm.

Data are represented mean ± SD. *P<0.05, **P<0.01, ***P<0.001.

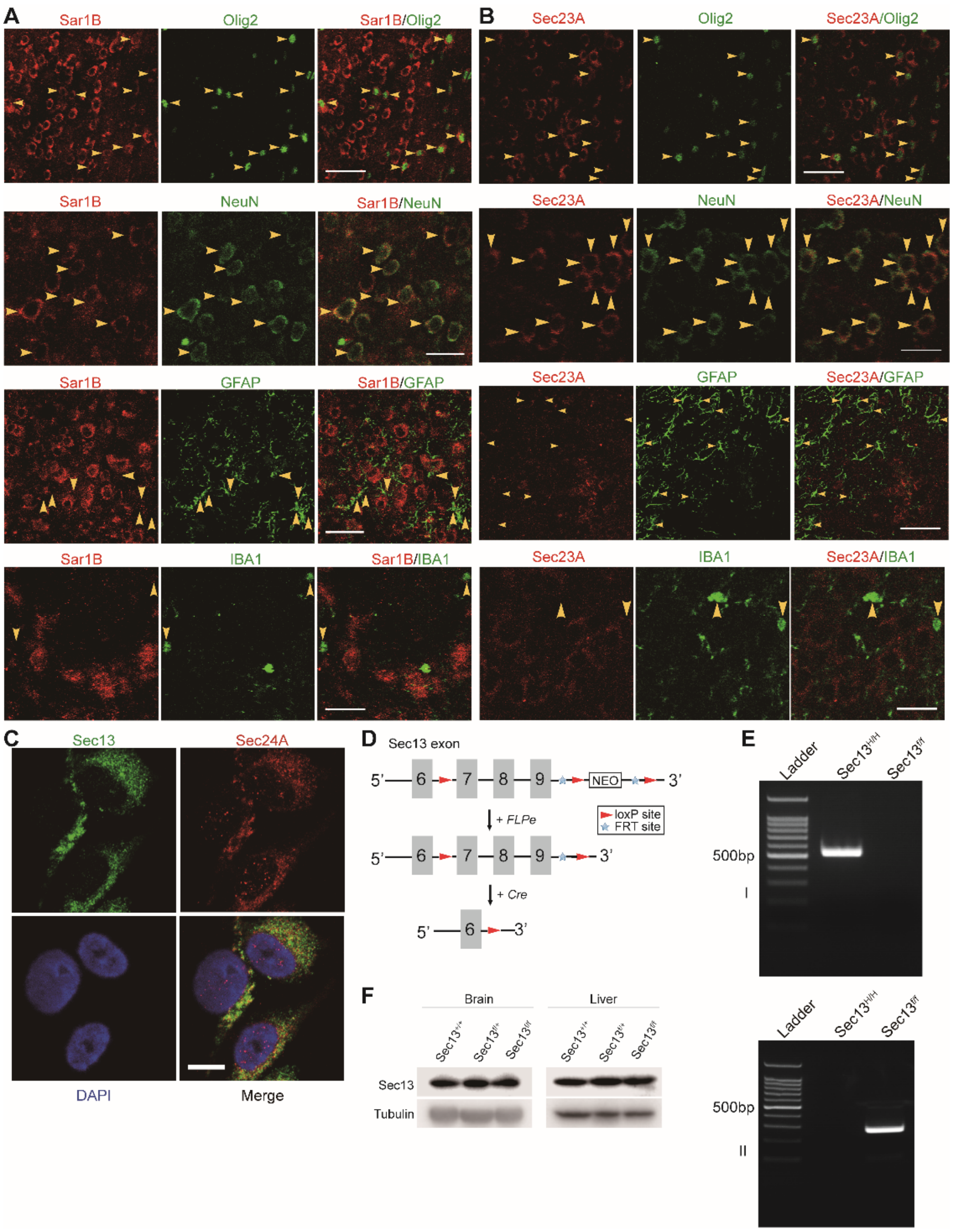

A
Sar1B
Olig2
Sar1B/Olig2
NeuN
Sar1B/NeuN
GFAP
Sar1B/GFAP
IBA1
Sar1B/IBA1
B
Sec23A
Sec23A/Olig2
Sec23A/NeuN
Sec23A/GFAP
Sec23A/IBA1
C
Sec13
Sec24A
DAPI
Merge
D
Sec13 exon
5'
6
7
8
9
NEO
3'
+ FLPe
loxP site
FRT site
+ Cre
E
Ladder
500bp
I
II
F
Brain
Liver
Sec13
Tubulin

Supplemental Figure 4. COPII components are expressed in neurons and oligodendrocytes, but not in astrocyte and microglia.

(A-B) Immunostaining of Sar1B (A) or Sec23A (B) with NeuN, GFAP and IBA1 in the cortex adjacent to corpus callosum (NeuN) or corpus callosum (GFAP/IBA1) of mice at P14. Scale bars represent 30 μm.

(C) Co-immunostaining of Sec13 with Sec24A in primary rat differentiating immature oligodendrocytes. Scale bars represent 10 μm.

(D) Schematic diagram depicting the strategy used to remove NEO cassette with FLPe-mediated excision and Cre-mediated excision of the floxed *Sec13*. LoxP sites flanking exon 7 and 9 of *Sec13* are indicated.

(E) Genotyping was performed with primer sets that detect: (I) the allele containing NEO cassette; (II) the allele deleted of NEO cassette.

(F) Immunoblotting of indicated proteins in brain and liver lysate from adult P120 wild-type, $Sec13^{flox/+}$, and $Sec13^{flox/flox}$ mice.

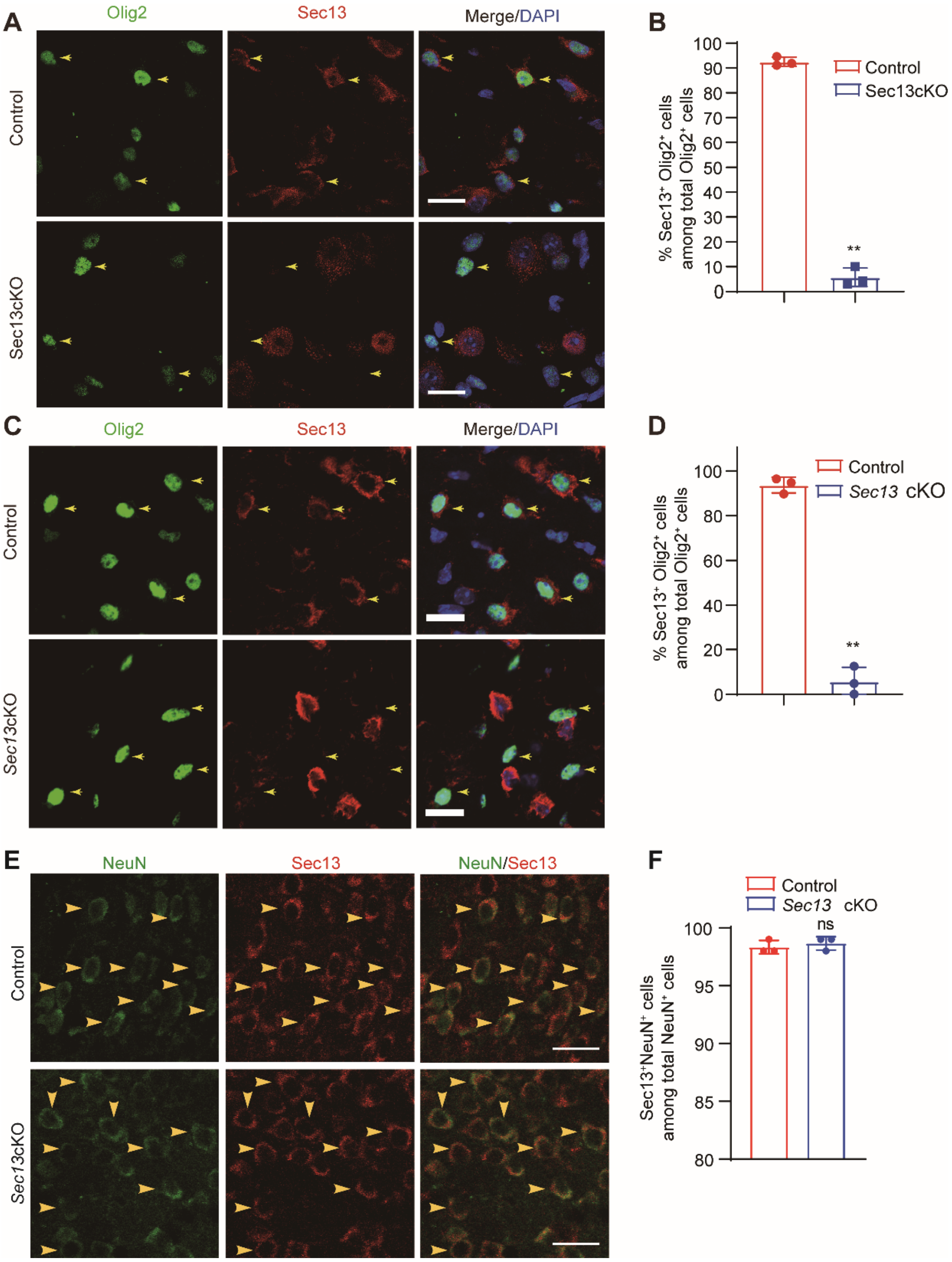
A
Olig2
Sec13
Merge/DAPI
Control
Sec13cKO
B
% Sec13+ Olig2+ cells among total Olig2+ cells
Control
Sec13cKO
**
C
Olig2
Sec13
Merge/DAPI
Control
Sec13cKO
D
% Sec13+ Olig2+ cells among total Olig2+ cells
Control
Sec13 cKO
**
E
NeuN
Sec13
NeuN/Sec13
Control
Sec13cKO
F
Sec13+NeuN+ cells among total NeuN+ cells
Control
Sec13 cKO
ns

Supplemental Figure 5. Sec13 is efficiently ablated in oligodendrocyte lineage.
(A) Immunostaining of Olig2 and Sec13 in the spinal cord of control and *Sec13*cKO mice at P14. Arrowheads indicate the $Olig2^+$ cells. Scale bars represent 50 μm.
(B) Quantification of $Sec13^+$ $Olig2^+$ cells as a percentage of total $Olig2^+$ cells in the spinal cord at P14 (n= 3 control and 3 mutant animals).
(C) Immunostaining of Olig2 and Sec13 in the corpus callosum of control and *Sec13*cKO mice at P14. Arrowheads indicate the $Olig2^+$ cells. Scale bars represent 20 μm.
(D) Quantification of $Sec13^+$ $Olig2^+$ cells as a percentage of total $Olig2^+$ cells in the corpus callosum at P14 (n= 3 control and 3 mutant animal).
(E) Immunostaining of NeuN and Sec13 in the cortex adjacent to corpus callosum of control and *Sec13*cKO mice at P14. Arrowheads indicate the $NeuN^+$/$Sec13^+$ cells. Scale bars represent 200 μm.
(F) Quantification of $Sec13^+$ $NeuN^+$ cells as a percentage of total $NeuN^+$ cells in the cortex adjacent to corpus callosum of control and mutant mice at P14 (n= 3 control and 3 mutant animal). Data are represented as mean ± SD; $^{**}P<0.01$, two-tailed unpaired Student's *t* test.

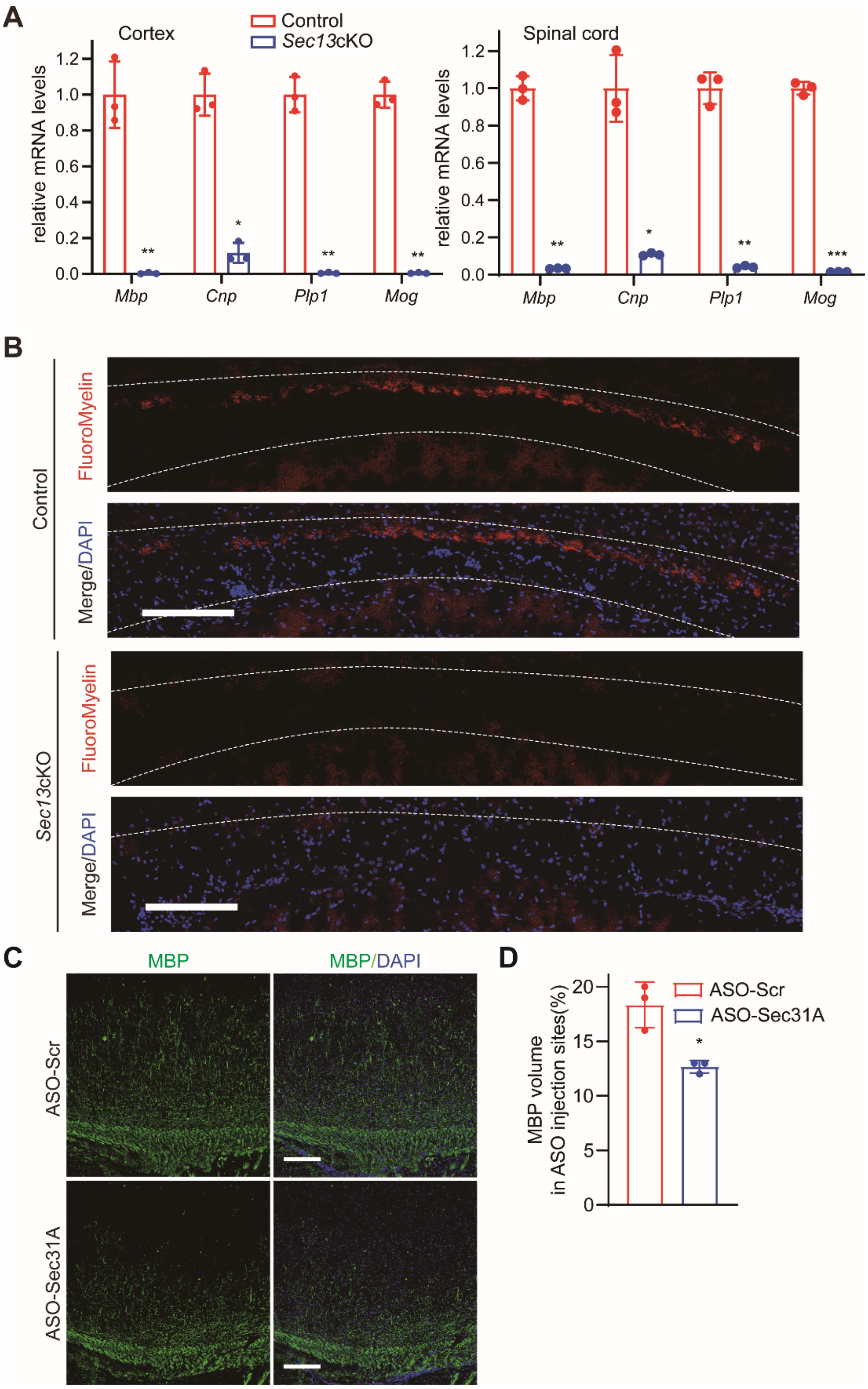

A
Cortex
Control
Sec13cKO
relative mRNA levels
Mbp
Cnp
Plp1
Mog
Spinal cord
B
Control
FluoroMyelin
Merge/DAPI
Sec13cKO
C
MBP
MBP/DAPI
ASO-Scr
ASO-Sec31A
D
MBP volume
in ASO injection sites(%)
ASO-Scr
ASO-Sec31A

Supplemental Figure 6. COPII components is required for myelination.

(A) Real-time PCR analysis of myelination-associated genes in cortex (left) and spinal cord (right) of control and *Sec13*cKO at P14 (n= 3 control and 3 mutant animals).

(B) Images of corpus callosum stained with FluoroMyelin-red in control and *Sec13*cKO at P14. Scale bars represent 200 μm.

(C) Immunostaining of MBP in corpus callosum and cortex of ASO-Scr and ASO-Sec31A injected mice at P14. Scr=scramble. Scale bars represent 200 μm.

(D) Quantification of MBP^+ volume in ASO-Scr and ASO-Sec31A injected mice (n= 3 animals/treatment). Data are represented as mean ± SD; $^*P<0.05$, $^{**}P<0.01$, $^{***}P<0.001$, two-tailed unpaired Student's *t* test.

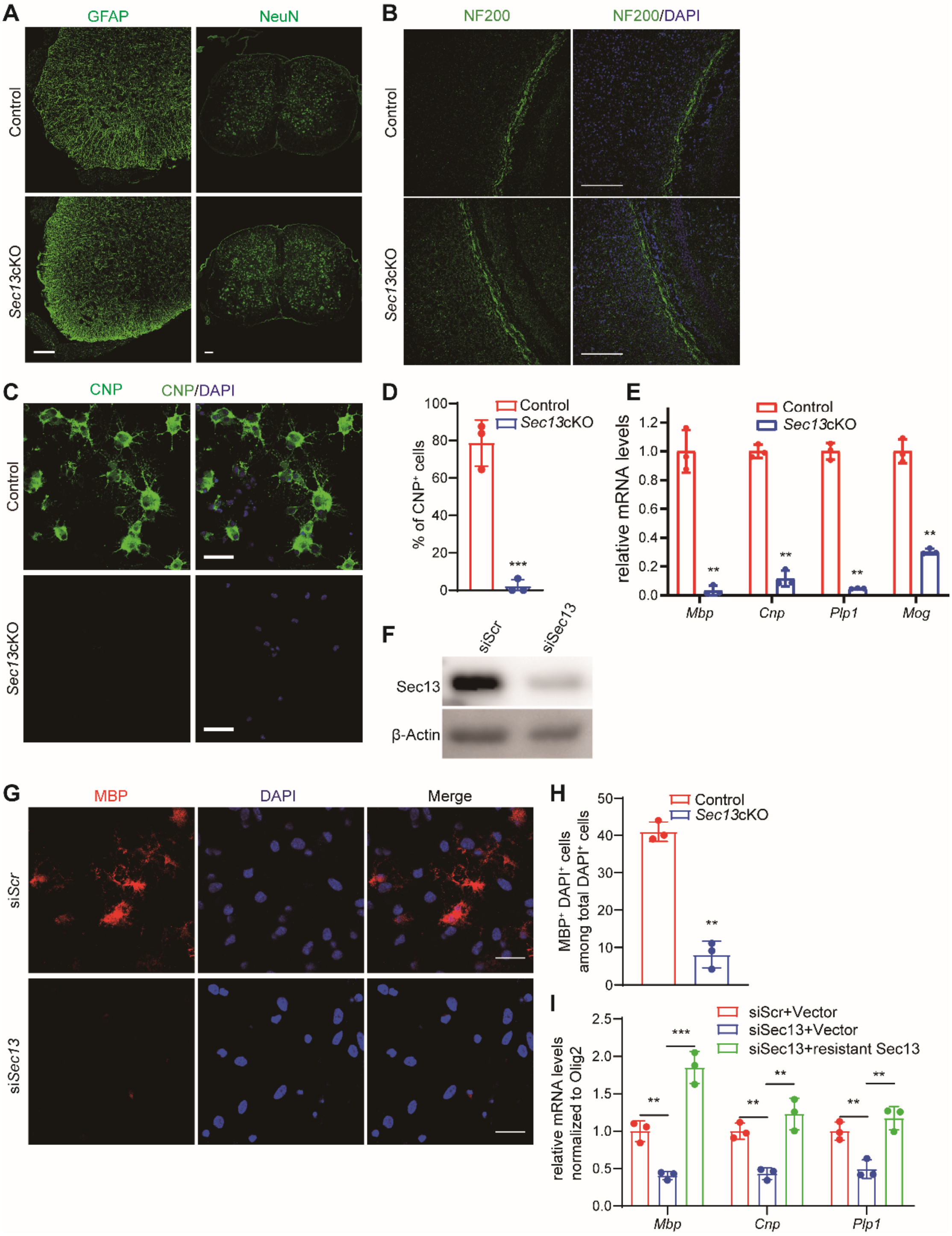
A
GFAP
NeuN
Control
Sec13cKO
B
NF200
NF200/DAPI
C
CNP
CNP/DAPI
D
% of CNP+ cells
Control
Sec13cKO
***
E
relative mRNA levels
Mbp
Cnp
Plp1
Mog
F
siScr
siSec13
Sec13
β-Actin
G
MBP
DAPI
Merge
siScr
siSec13
H
MBP+ DAPI+ cells among total DAPI+ cells
I
relative mRNA levels normalized to Olig2
siScr+Vector
siSec13+Vector
siSec13+resistant Sec13

Supplemental Figure 7. Sec13 deletion in the OL lineage does not affect the development of other neural cell types in the brain, but inhibits OL differentiation. (A-B) Immunostaining of astrocyte marker GFAP, neuron marker NeuN in the spinal cords of control and *Sec13*cKO at P14. Scale bars represent 100 μm.

(B) Immunostaining of NF200 in the corpus callosum of control and *Sec13*cKO mice at P14. Scale bars represent 200 μm.

(C-D) Immunolabeling (C) and quantification (D) of CNP in control and *Sec13*cKO primary OPCs under differentiation conditions for 96h (n= 3 animals/treatment). Scale bars represent 50 μm. Data were analyzed two-tailed unpaired Student's *t* test.

(E) Real-time PCR analysis of myelination-associated genes in control and *Sec13*cKO primary OPCs under differentiation conditions (n= 3 independent experiments). Data were analyzed two-tailed unpaired Student's *t* test.

(F) Immunoblotting validation of knockdoown efficiency of Sec13 in primary rat OPCs treated with scrambled or *Sec13* siRNAs.

(G-H) Immunolabeling (G) and quantification (H) of MBP in primary rat OPCs under differentiation conditions for 72hours following treatments with scrambled or *Sec13* siRNAs (n= 3 independent experiments). Scale bars represent 25 μm. SCR, scrambled.

(I) Real-time PCR analysis of myelination-associated genes in primary rat OPCs under differentiation conditions for 48hours following treatments with scrambled or *Sec13* siRNAs with control vector or vectors overexpressing resistant Sec13 (n= 3 independent experiments. Data were analyzed by 1-way ANOVA with Tukey's correction for multiple comparisons. Data are represented as mean ± SD; *P<0.05, **P<0.01, ***P<0.001.

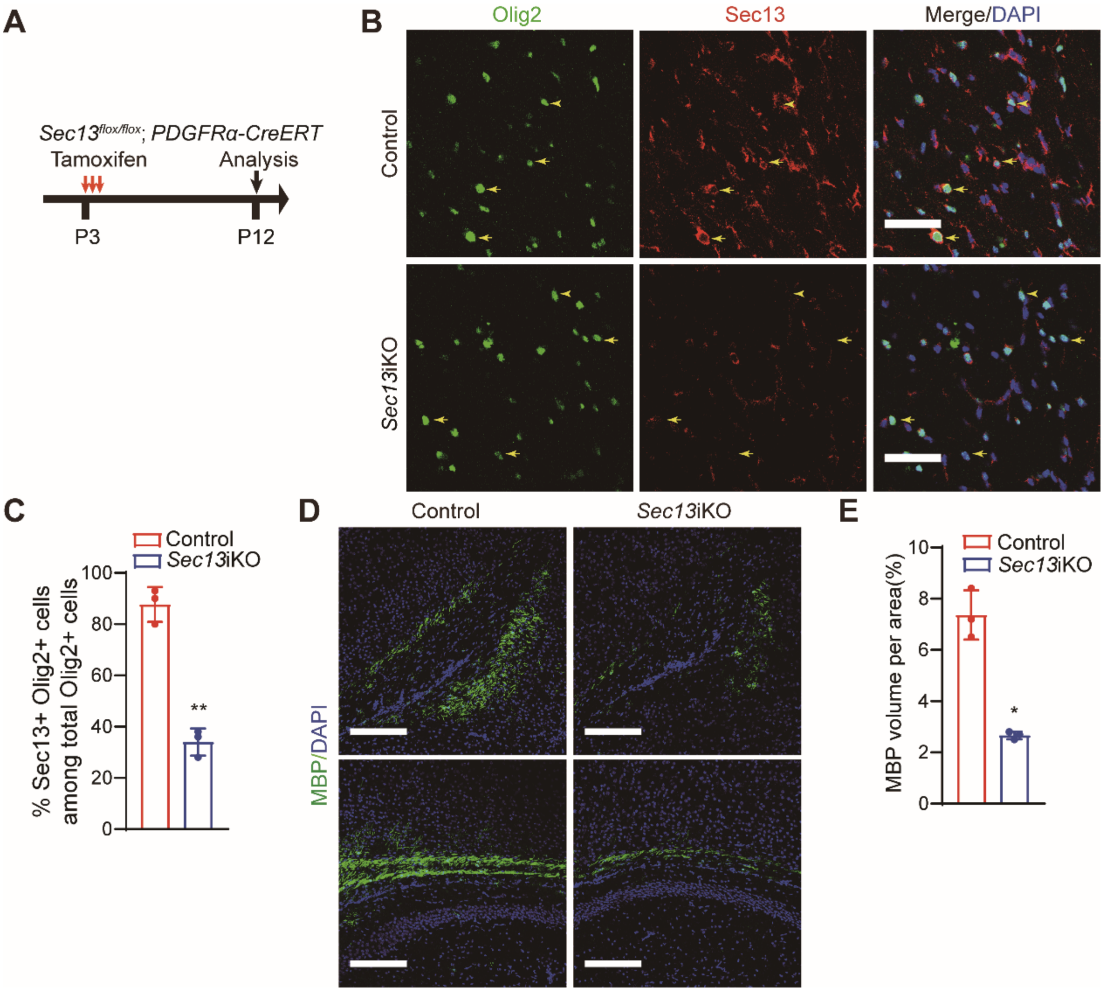
A
Sec13flox/flox; PDGFRα-CreERT
Tamoxifen
Analysis
P3
P12
B
Olig2
Sec13
Merge/DAPI
Control
Sec13iKO
C
Control
Sec13iKO
% Sec13+ Olig2+ cells among total Olig2+ cells
100
80
60
40
20
0
**
D
Control
Sec13iKO
MBP/DAPI
E
Control
Sec13iKO
MBP volume per area(%)
10
8
6
4
2
0
*

Supplemental Figure 8. Sec13 is required for myelination in CNS.

(A) Diagram showing tamoxifen administration to induce *Sec13* deletion.

(B) Immunostaining of Olig2 and Sec13 in the corpus callosum of control and *Sec13*iKO mice at P12. Scale bars represent 50 μm.

(C) Quantification of $Sec13^{+}Olig2^{+}$ cells as a percentage of total $Olig2^{+}$ cells in the corpus callosum of control and *Sec13*iKO mice at P12 (n= 3 control and 3 mutant animals).

(D-E) Immunostaining (D) and quantification (E) of MBP in the corpus callosum of control and *Sec13*iKO mice at P12 (n= 3 control and 3 mutant animals). Scale bars represent 200 μm. Data are represented mean ± SD; $^{*}P<0.05$, $^{**}P<0.01$, two-tailed unpaired Student's t test.

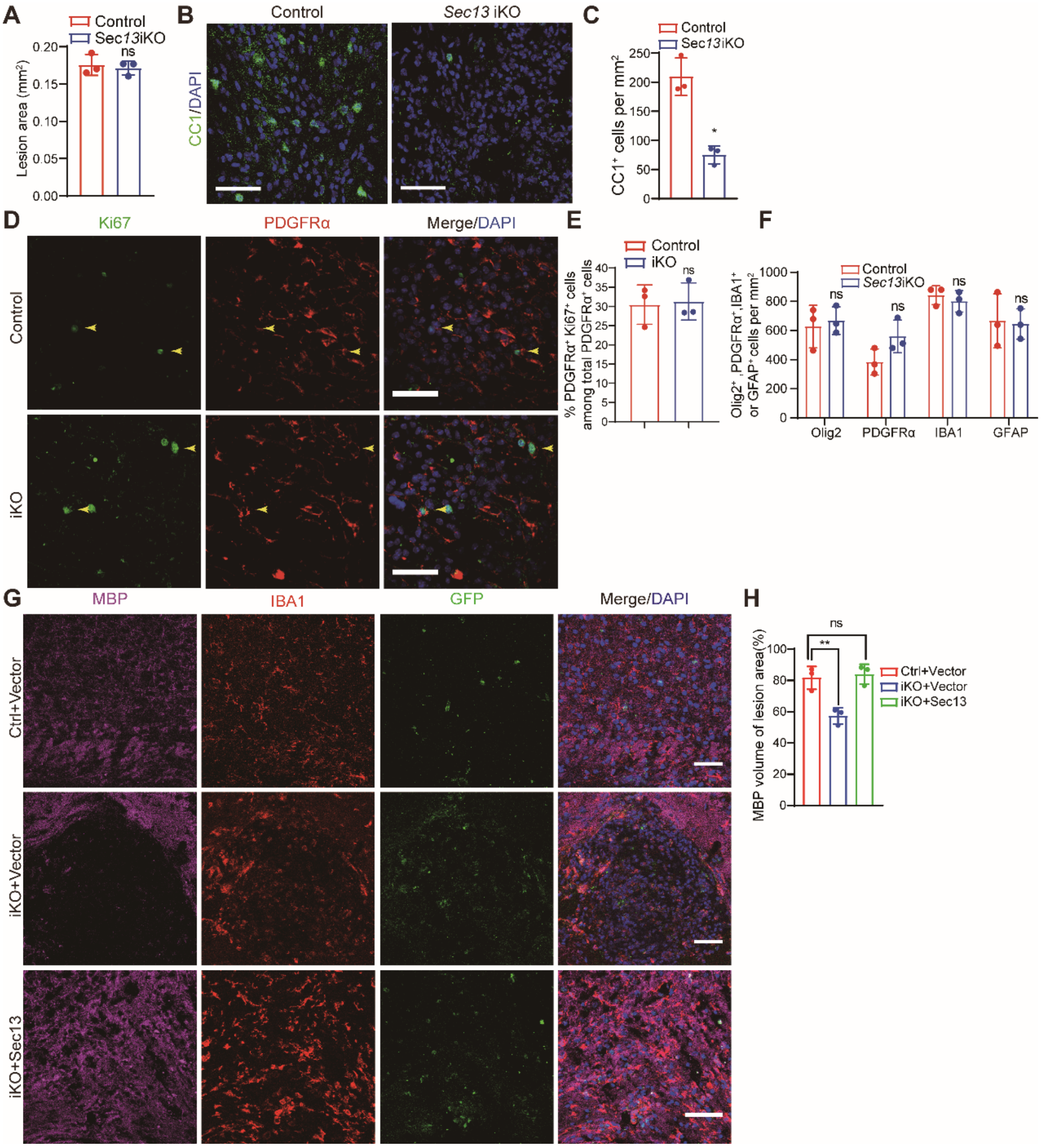

A
Control
Sec13iKO
ns
Lesion area (mm2)
0.20
0.15
0.10
0.05
0.00
B
Control
Sec13 iKO
CC1/DAPI
C
Control
Sec13iKO
CC1+ cells per mm2
250
200
150
100
50
0
*
D
Ki67
PDGFRα
Merge/DAPI
Control
iKO
E
Control
iKO
ns
% PDGFRα+ Ki67+ cells among total PDGFRα+ cells
40
35
30
25
20
15
10
5
0
F
Control
Sec13iKO
ns
Olig2+, PDGFRα+, IBA1+ or GFAP+ cells per mm2
1000
800
600
400
200
0
Olig2
PDGFRα
IBA1
GFAP
G
MBP
IBA1
GFP
Merge/DAPI
Ctrl+Vector
iKO+Vector
iKO+Sec13
H
ns
**
MBP volume of lesion area(%)
100
80
60
40
20
0
Ctrl+Vector
iKO+Vector
iKO+Sec13

Supplemental Figure 9. Sec13 is critical for adult remyelination after demyelination.

(A) Quantification of lesion area in corpus callosum lesions of control and *Sec13*iKO mice at 14 dpl (n= 3 control and 3 mutant animals). Data were analyzed two-tailed unpaired Student's *t* test.

(B and C) Immunostaining (B) and quantification (C) of $CC1^+$ cells in corpus callosum lesions of control and *Sec13*iKO mice at 14 dpl (n= 3 control and 3 mutant animals). Scale bars represent 50 μm. Data were analyzed two-tailed unpaired Student's *t* test.

(D) Immunostaining of Ki67 and PDGFRα in corpus callosum lesions of control and *Sec13* iKO mice at 14 dpl. Arrowheads indicate the $Ki67^+PDGFR\alpha^+$ cells. Scale bars represent 50 μm.

(E) Quantification of $PDGFR\alpha^+$ $Ki67^+$ cells as a percentage of total $PDGFR\alpha^+$ cells in the corpus callosum lesions of control and *Sec13* iKO mice at 14 dpl (n= 3 control and 3 mutant animals). Data were analyzed two-tailed unpaired Student's *t* test.

(F) Quantification of $Olig2^+$, $PDGFR\alpha^+$, $IBA1^+$, and $GFAP^+$ cells in the corpus callosum lesions of control and *Sec13*iKO at 14 dpl (n= 3 control and 3 mutant animals). Data were analyzed two-tailed unpaired Student's *t* test.

(G) Immunostaining of MBP, IBA1, and GFP in corpus callosum lesions of control and *Sec13* iKO mice injected with lentivirus expressing GFP or Sec13-GFP at 14 dpl. Lentivirus and LPC were focal injected at the same time. Scale bars represent 50 μm.

(H) Quantification of MBP^+ volume in corpus callosum lesions of control and *Sec13* iKO mice injected with lentivirus expressing GFP or Sec13-GFP at 14 dpl (n= 3 animals/treatment). Data were analyzed by 1-way ANOVA with Tukey's correction for multiple comparisons.Data are represented mean ± SD; $^{*}P<0.05$, $^{**}P<0.01$.

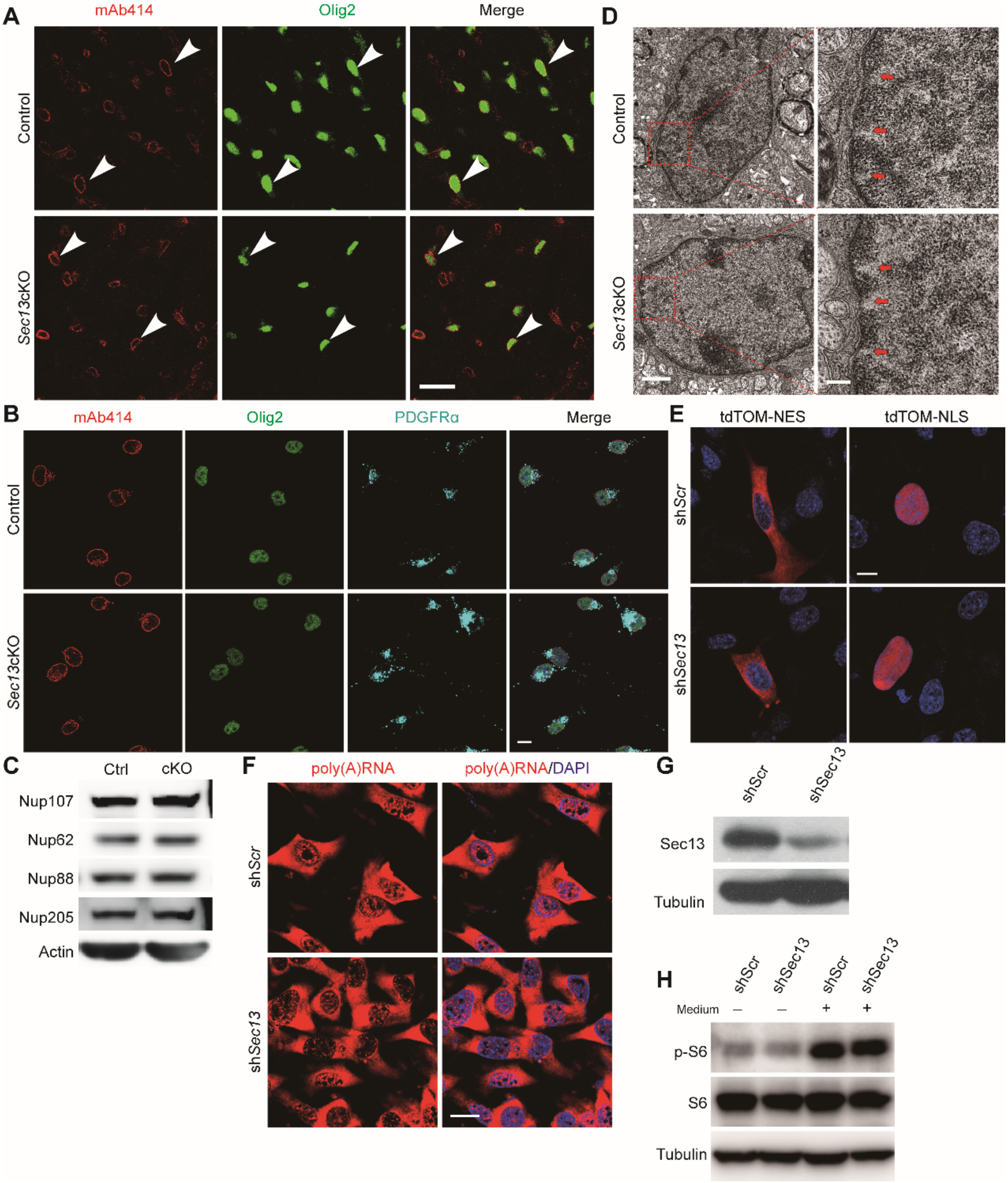
A
mAb414
Olig2
Merge
Control
Sec13cKO
B
mAb414
Olig2
PDGFRα
Merge
Control
Sec13cKO
C
Ctrl
cKO
Nup107
Nup62
Nup88
Nup205
Actin
D
Control
Sec13cKO
E
tdTOM-NES
tdTOM-NLS
shScr
shSec13
F
poly(A)RNA
poly(A)RNA/DAPI
shScr
shSec13
G
shScr
shSec13
Sec13
Tubulin
H
shScr
shSec13
shScr
shSec13
Medium
–
–
+
+
p-S6
S6
Tubulin

Supplemental Figure 10. Ablation of Sec13 does not affect nucleocytoplasmic transport.

(A) Immunostaining of mAb414 and Olig2 in spinal cord of P14 control and *Sec13*cKO mice. Arrowheads indicate mAb414$^+$/ Olig2$^+$ cells. Scale bars represent 25 μm.

(B) Immunostaining of mAb414, Olig2, and PDGFRα in primary control and *Sec13*cKO OPCs. Scale bars represent 10 μm.

(C) Immunoblotting of indicated proteins in the spinal cords lysate of control and *Sec13*cKO at P7. Ctrl: control; cKO: *Sec13*cKO.

(D) Electron microscopy images of oligodendrocytes from control and *Sec13*cKO optic nerves. Boxed image is shown in the right. Arrows indicate NPCs. Scale bars represent 1 μm in left panel and 0.2 μm in right panel.

(E) Immunofluorescence of tdTomato-NES and tdTomato-NLS signals in Oli-neu cells transduced with scrambled or *Sec13* shRNA. NES, nuclear export signal; NLS, nuclear localization signal. Nuclei are stained with DAPI. Scale bars represent 10 μm.

(F) Oligo-dT *In situ* hybridization followed by fluorescence microscopy in Oli-neu cells transduced with scrambled or *Sec13* shRNA. Nuclei are stained with DAPI. Scale bars represent 20 μm.

(G) Immunoblotting validation of knockdown efficiency of Sec13 in Oli-neu cells. shSCR, scrambled shRNA; shSec13, *Sec13* shRNA.

(H) Immunoblotting of indicated proteins in Oli-neu cells transduced with scrambled or *Sec13* shRNA following amino acid-induced activation of mTORC1.

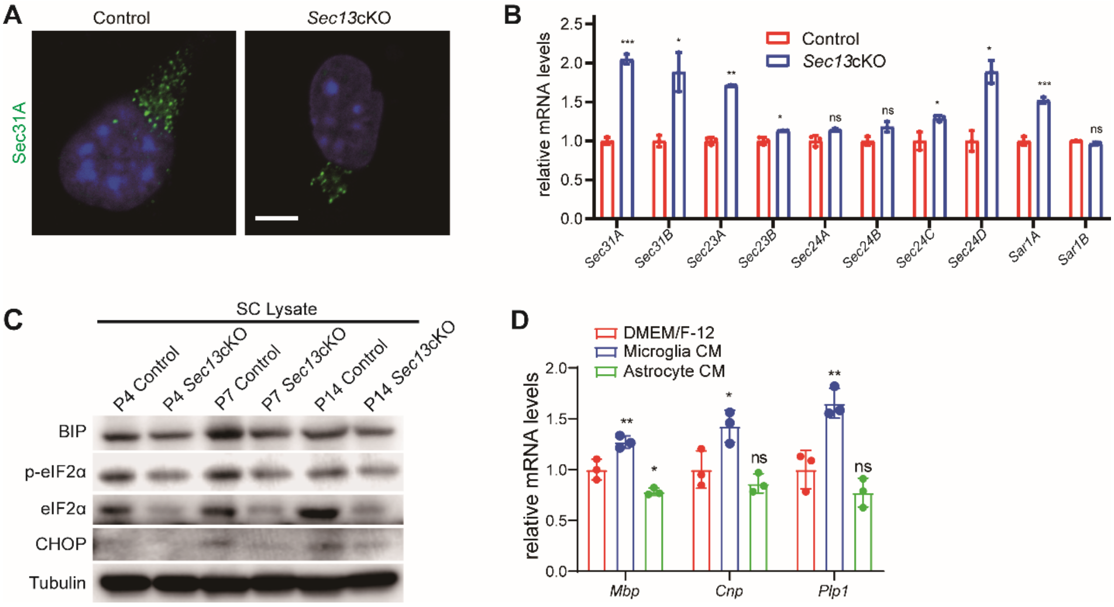

Supplemental Figure 11. Loss of Sec13 affect other COPII components.

(A) Immunostaining of Sec31A in control and *Sec13*cKO primary OPCs. Scale bars represent 5 μm.

(B) Real-time PCR analysis of COPII components in control and *Sec13*cKO primary OPCs (n=3 independent experiments). Data were analyzed by two-tailed unpaired Student's *t* test.

(C) Immunoblotting of indicated proteins in spinal cord lysate of control and *Sec13*cKO at P4, P7, and P14.

(D) Real-time PCR analysis of myelination-associated genes in primary rat OPCs under differentiation conditions following treatments with DMEM/F-12 or microglia CM or astrocyte CM (n=3 independent experiments). Data were analyzed by 1-way ANOVA with Tukey's correction for multiple comparisons. Data are represented mean ± SD; **P*<0.05, ***P*<0.01, ****P*<0.001.

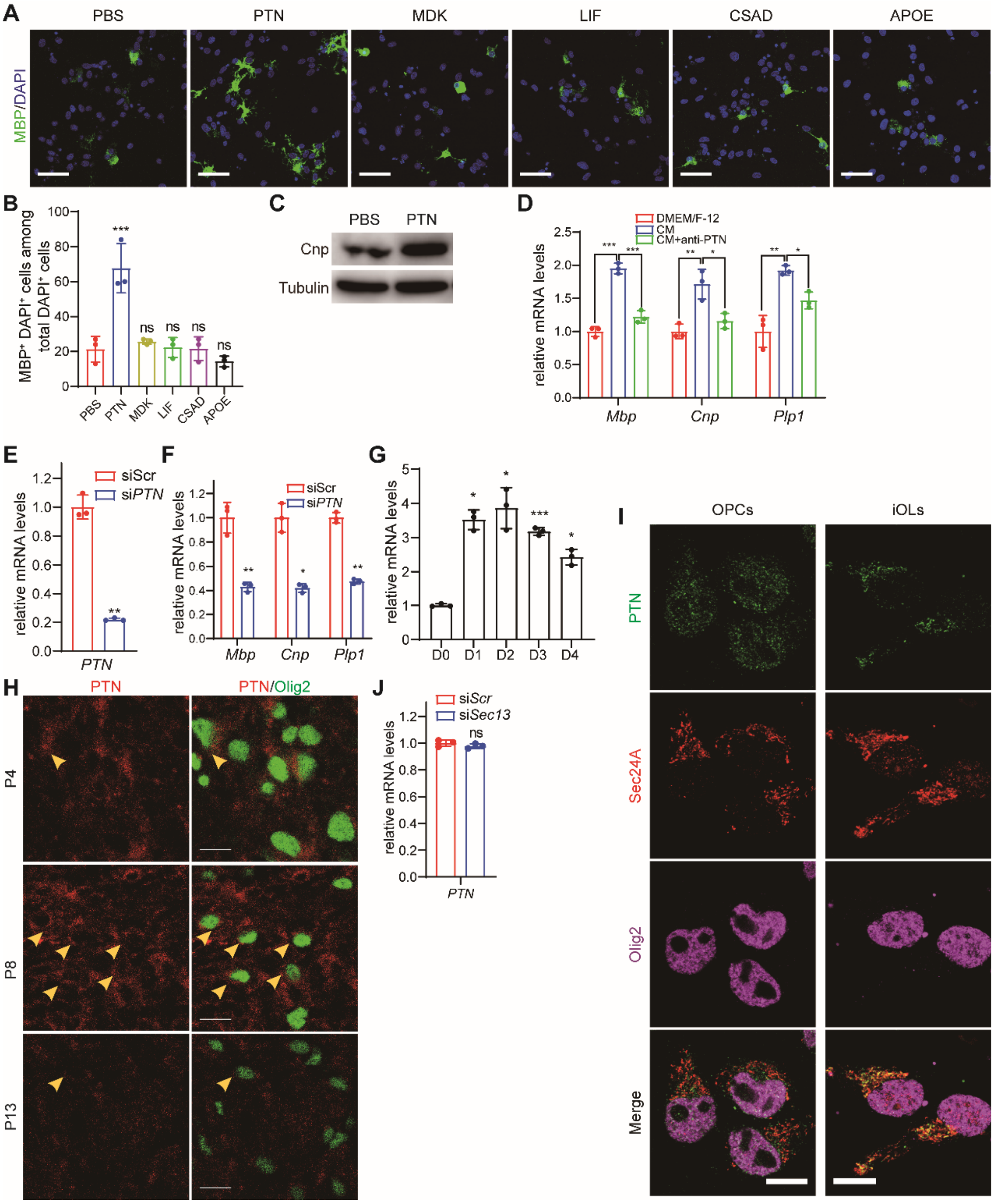
A
PBS
PTN
MDK
LIF
CSAD
APOE
MBP/DAPI
B
MBP⁺ DAPI⁺ cells among total DAPI⁺ cells
***
ns
C
PBS
PTN
Cnp
Tubulin
D
DMEM/F-12
CM
CM+anti-PTN
relative mRNA levels
Mbp
Cnp
Plp1
E
siScr
siPTN
PTN
F
siScr
siPTN
G
D0
D1
D2
D3
D4
H
PTN
PTN/Olig2
P4
P8
P13
I
OPCs
iOLs
PTN
Sec24A
Olig2
Merge
J
siScr
siSec13
ns

Supplemental Figure 12. PTN promotes OPC differentiation.

(A-B) Immunolabeling (A) and quantification (B) of MBP in primary rat OPCs under differentiation conditions without T3 for 96 hours following treatments with indicated recombinant proteins (n= 3 independent experiments). Scale bars represent 50 μm.

(C) Immunoblotting of indicated proteins in primary rat OPCs treated with recombinant PTN.

(D) Real-time PCR analysis of myelination-associated genes in primary rat OPCs under differentiation conditions following treatments with DMEM/F-12, or CM, or CM depleted of PTN via immunoprecipitation with anti-PTN antibody (n= 3 independent experiments).

(E) Real-time PCR analysis of *PTN* in primary rat OPCs treated with scrambled or *PTN* siRNAs (n=3 independent experiments).

(F) Real-time PCR analysis of myelination-associated genes in primary rat OPCs under differentiation conditions following treatment with scrambled or *PTN* siRNAs (n=3 independent experiments).

(G) Real-time PCR analysis of *PTN* in primary rat OPCs and differentiating oligodendrocytes after T3 treatment. D, day (n=3 independent experiments).

(H) Immunostaining of PTN with Olig2 in corpus callosum of wild-type mice at P4, P8, and P13. Scale bars represent 30 μm.

(I) Immunostaining of PTN with Olig2 and Sec24A in primary rat OPCs (left) or differentiating immature oligodendrocytes (iOLs, right). Scale bars represent 10 μm.

(J) Real-time PCR analysis of *PTN* in primary rat OPCs treated with scrambled or *Sec13* siRNAs (n=3 independent experiments).

(E, F and J) Data were analyzed by two-tailed unpaired Student's *t* test. (B, D and G) Data were analyzed by 1-way ANOVA with Tukey's correction for multiple comparisons. Data are represented mean ± SD; $^{*}P$<0.05, $^{**}P$<0.01, $^{***}P$<0.001.

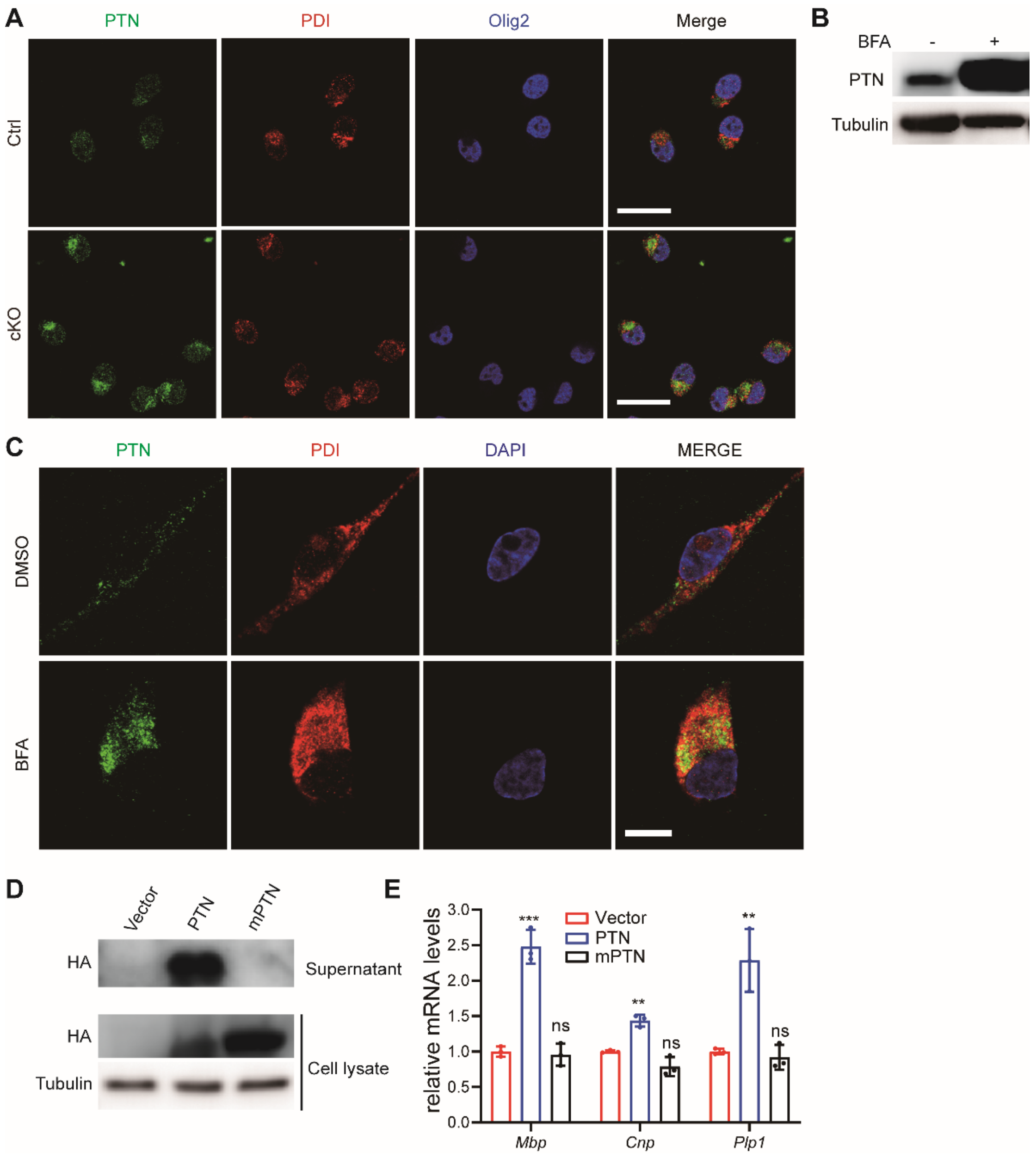

A
PTN
PDI
Olig2
Merge
Ctrl
cKO
B
BFA
-
+
PTN
Tubulin
C
PTN
PDI
DAPI
MERGE
DMSO
BFA
D
Vector
PTN
mPTN
HA
Supernatant
HA
Tubulin
Cell lysate
E
relative mRNA levels
Vector
PTN
mPTN
***
**
**
ns
ns
ns
Mbp
Cnp
Plp1

Supplemental Figure 13. PTN is secreted through COPII regulation.

(A) Immunostaining of PTN, PDI, and Olig2 in primary control and *Sec13*cKO differentiating immature oligodendrocytes. Scale bars represent 20 μm.

(B) Immunoblotting of indicated proteins in primary rat OPCs treated with BFA (0.5 μg/μL) for 24h.

(C) Immunostaining of PTN and PDI in primary rat differentiating immature oligodendrocytes in the presence or absence of BFA (0.5 μg/μL) for 24h. Scale bars represent 10 μm.

(D) Immunoblotting of indicated proteins in culture medium (upper, supernatant) and cell lysate (lower) from primary rat OPCs transfected with HA-PTN or HA-PTN mutant (L18&20R) construct.

(E) Real-time PCR analysis of myelination-associated genes in primary rat OPCs under differentiation conditions following PTN or PTN mutant (L18&20R) over expression (n=3 independent experiments). Data were analyzed by 1-way ANOVA with Tukey's correction for multiple comparisons. Data are represented mean ± SD; $^{**}P<0.01$, $^{***}P<0.001$.

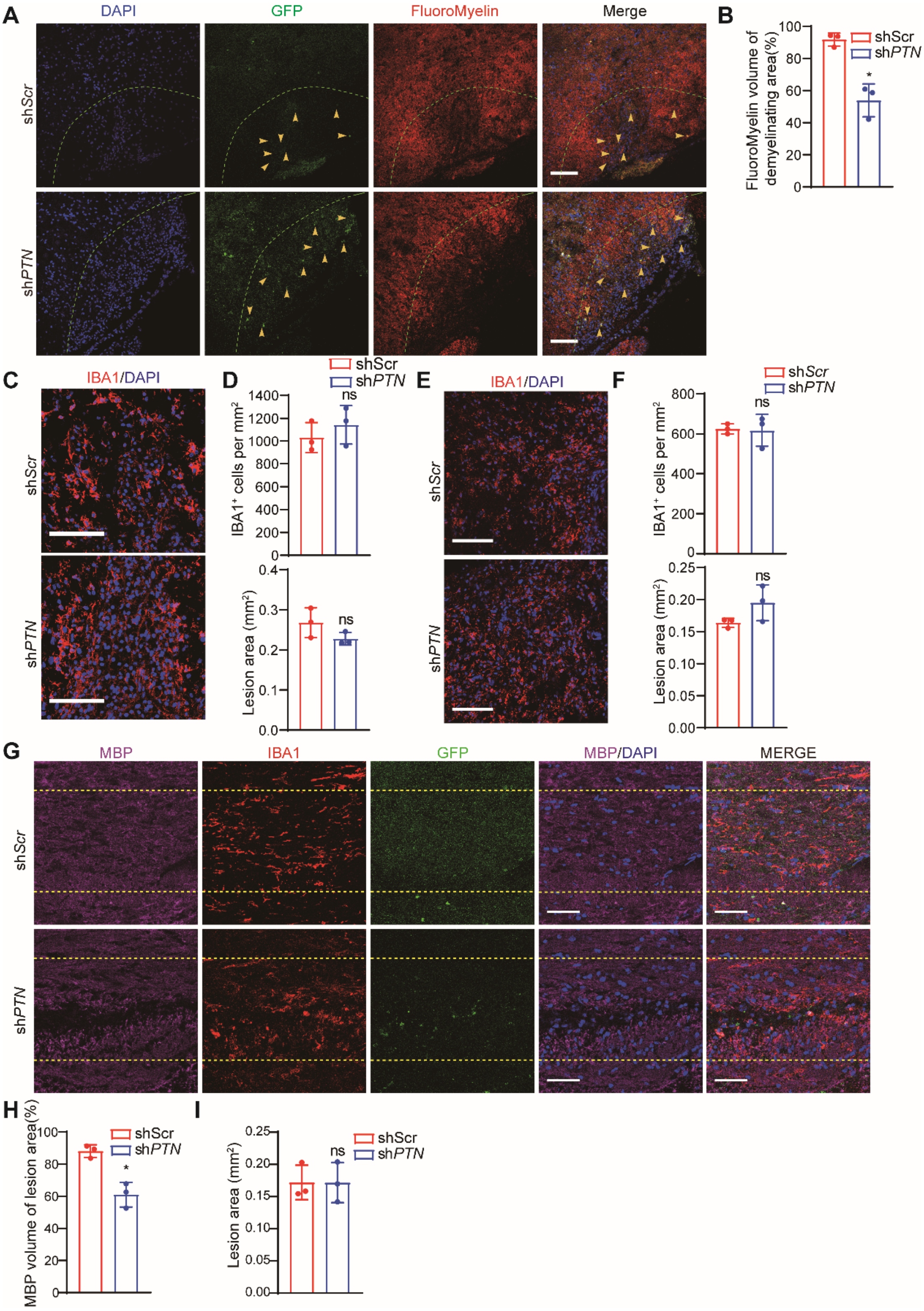
A
DAPI
GFP
FluoroMyelin
Merge
shScr
shPTN
B
FluoroMyelin volume of demyelinating area(%)
shScr
shPTN
*
C
IBA1/DAPI
shScr
shPTN
D
shScr
shPTN
ns
IBA1+ cells per mm2
Lesion area (mm2)
E
IBA1/DAPI
shScr
shPTN
F
shScr
shPTN
ns
IBA1+ cells per mm2
Lesion area (mm2)
G
MBP
IBA1
GFP
MBP/DAPI
MERGE
shScr
shPTN
H
MBP volume of lesion area(%)
shScr
shPTN
*
I
Lesion area (mm2)
shScr
shPTN
ns

Supplemental Figure 14. Loss of PTN impaired remyelination.

(A) Representative images of GFP and FluoroMyelin in spinal cord lesions of mice after injection of lentivirus expressing scrambled or *PTN* shRNA at 21 dpl. Arrowheads indicated GFP^+ cells. Scale bars represent 100 μm.

(B) Quantification of FluoroMyelin volume in spinal cord lesions of mice injected with lentivirus expressing scrambled or *PTN* shRNA at 21 dpl (n= 3 animals/treatment).

(C) Immunostaining of IBA1 in spinal cord lesions of mice injected with lentivirus expressing scrambled or *PTN* shRNA at 14 dpl. Scale bars represent 100 μm.

(D) Quantification of $IBA1^+$ cells (left panel) and lesion area (right panel) in spinal cord lesions of mice injected with lentivirus expressing scrambled or *PTN* shRNA at 14 dpl (n= 3 animals/treatment).

(E) Immunostaining of IBA1 in spinal cord lesions of mice injected with lentivirus expressing scrambled or *PTN* shRNA at 21 dpl. Scale bars represent 100 μm.

(F) Quantification of $IBA1^+$ cells (left panel) and lesion area (right panel) in spinal cord lesions of mice injected with lentivirus expressing scrambled or *PTN* shRNA at 21 dpl (n= 3 animals/treatment).

(G) Representative images of MBP, IBA1, and GFP in corpus callosum lesions of mice after injection of lentivirus expressing scrambled or *PTN* shRNA at 14 dpl. Scale bars represent 50 μm.

(H) Quantification of MBP volume in corpus callosum lesions of mice after injection of lentivirus expressing scrambled or *PTN* shRNA at 14 dpl (n= 3 animals/treatment).

(I) Quantification of lesion area in corpus callosum lesions of mice after injection of lentivirus expressing scrambled or *PTN* shRNA at 14 dpl (n= 3 animals/treatment). Data represent mean±SD; $^*P < 0.05$; two-tailed unpaired Student's *t* test.

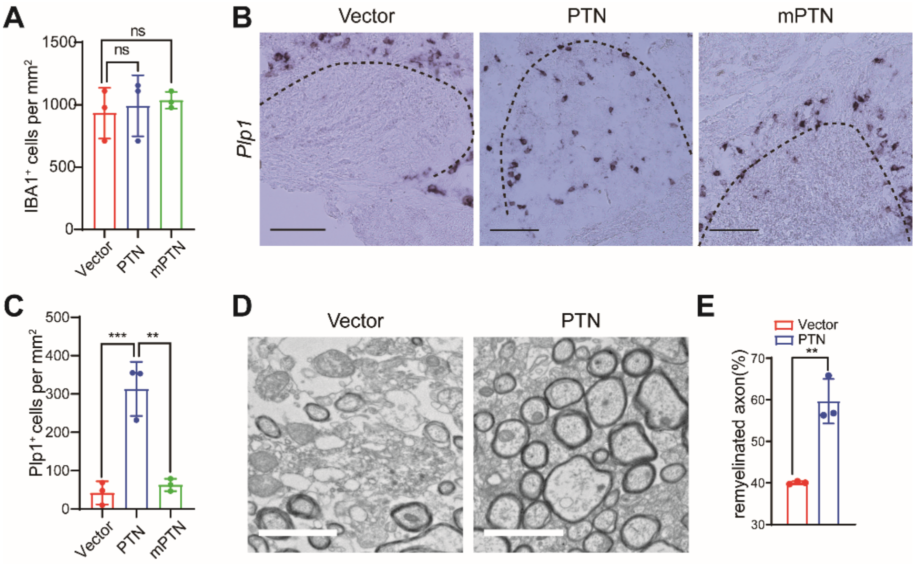

Supplemental Figure 15. PTN accelerate remyelination after demyelinating injury.
(A) Quantification of IBA1^{+} cells in corpus callosum lesions of mice after injection of retrovirus expressing PTN or PTN mutant (L18&20R) at 10 dpl (n= 3 animals/treatment). Data were analyzed by 1-way ANOVA with Tukey's correction for multiple comparisons.
(B-C) *In situ* hybridization (B) and quantification (C) of *Plp1* in the spinal cord LPC lesions of mice after injection of retrovirus expressing PTN or PTN mutant (L18&20R) at 10 dpl (n= 3 animals/treatment). Scale bars represent 100 μm. Data were analyzed by 1-way ANOVA with Tukey's correction for multiple comparisons.
(D) Electron microscopy of LPC lesion from corpus callosum of mice after injection of retrovirus expressing PTN at 10 dpl. Scale bars represent 2 μm.
(E) Quantification of remyelinated axons in LPC-induced lesion in corpus callosum of mice after injection of retrovirus expressing PTN at 10 dpl (n=3 animals/treatment). Data were analyzed by two-tailed unpaired Student's *t* test. Data are represented mean ± SD; $^{**}P<0.01$, $^{***}P<0.001$.
